# External communication of automated shuttles: Results, experiences, and lessons learned from three European long-term research projects

**DOI:** 10.3389/frobt.2022.949135

**Published:** 2022-10-26

**Authors:** Alexander G. Mirnig, Magdalena Gärtner, Peter Fröhlich, Vivien Wallner, Anna Sjörs Dahlman, Anna Anund, Petr Pokorny, Marjan Hagenzieker, Torkel Bjørnskau, Ole Aasvik, Cansu Demir, Jakub Sypniewski

**Affiliations:** ^1^ Human-Computer Interaction Division, Department of Artificial Intelligence and Human Interfaces, University of Salzburg, Salzburg, Austria; ^2^ Center for Technology Experience, AIT Austrian Institute of Technology GmbH, Vienna, Austria; ^3^ Swedish National Road and Transport Research Institute, Linköping, Sweden; ^4^ Electrical Engineering, Chalmers University of Technology, Gothenburg, Sweden; ^5^ Rehabilitation Medicine, Linköping University, Linköping, Sweden; ^6^ Stockholm Stress Centre, Stockholm University, Stockholm, Sweden; ^7^ TØI—Institute of Transport Economics, Oslo, Norway

**Keywords:** automated shuttles, eHMI, user studies, shuttle2vehicle communication, shuttle2pedestrian communication

## Abstract

Automated shuttles are already seeing deployment in many places across the world and have the potential to transform public mobility to be safer and more accessible. During the current transition phase from fully manual vehicles toward higher degrees of automation and resulting mixed traffic, there is a heightened need for additional communication or external indicators to comprehend automated vehicle actions for other road users. In this work, we present and discuss the results from seven studies (three preparatory and four main studies) conducted in three European countries aimed at investigating and providing a variety of such external communication solutions to facilitate the exchange of information between automated shuttles and other motorized and non-motorized road users.

## 1 Introduction

Vehicle automation is considered a crucial aspect of “Vision Zero”, that is, the aim to achieve a state where there are no longer on-road accidents involving vehicles with fatal consequences. The efforts to automate mobility encompass both private and public means of transport, with automated shuttles being one of the currently more prominent facets of the latter, exploring not only automated mobility in terms of safety but also new mobility patterns, for example, mobility on demand.

Shuttles are, in essence, buses with smaller passenger capacities, which make them suitable for a variety of contexts (urban city centers or other areas with high amounts of pedestrian traffic, airports, or rural areas with lower demand in terms of number of passengers). These contexts are characterized by different traffic conditions and subsequent requirements when compared to contexts with higher volumes of motorized vehicles and higher speed limits (e.g., motorways, highly frequented roads, city peripheries, and a.s.o). Since other road users are either less frequent (especially in last mile or airport contexts) or simply a lot slower and/or pose less of a threat (pedestrians, cyclists, and scooters), such contexts could already see the deployment of (low velocity) automated shuttles, even without the technology being fully realized, due to these different circumstances and lower risk of accidents.

The transition to full vehicle automation is not yet complete and will not be for some time (e.g., Sheffi, 2015; Mraz, 2017; Lubitz, 2020). During this transition time, there is an increased need for clear communication of these vehicles with their (non-automated) environment, since the technology often responds differently to actions and maneuvers than a human would and there is no human behind the wheel that could serve as a fallback when a miscommunication or conflict occurs. Once automated vehicles are commonplace across traffic contexts and common interaction patterns have been established, these additional communication requirements are likely to diminish accordingly, although might not disappear entirely, especially in terms of fallback communication and conflict resolution. Since automated shuttles have now already seen deployment for several years and in a variety of contexts, there are already a good number of results and lessons learned to determine the way forward in terms of communication of automated shuttles with their traffic environment.

In this study, we collect and present the results from a series of studies concerning communication of automated shuttles with other road users. This study is a collaborative effort between three automated shuttle projects: the Austrian national flagship projects *auto.Bus—Seestadt* and *Digibus® Austria*, and the Horizon 2020 European project *Drive2TheFuture*. We present conceptual and field evaluations of interaction designs for communicating with motorized and non-motorized road users and draw results and design recommendations with regard to the complexity level of the information presented for both of these communication contexts.

## 2 Related work

The advent of automated vehicles has created a gap in communication of intent, which was usually maintained mainly *via* gestures and eye contact between human drivers (Rasouli et al., 2017; Kaleefathullah et al., 2020). Whether external human–machine interfaces (eHMI) can compensate for the lack of this communication is yet to be decided (e.g., Löcken et al., 2019; Kaleefathullah et al., 2020; De Clercq et al., 2019; Velasco et al., 2019). Several studies suggest that eHMIs can influence the confidence, trust, or perceived safety of crossing pedestrians (e.g., Löcken et al., 2019; Kaleefathullah et al., 2020; Kooijman et al., 2019; Velasco et al., 2019; De Clercq et al., 2019; Rettenmaier et al., 2020; Faas et al., 2021).

On the opposite side of the argument, there has been evidence suggesting that other road users base their decisions mostly on the implicit communication with the automated vehicles through its actions (e.g. Clamann et al., 2017; Palmeiro et al., 2018) Rettenmaier et al., 2019; Dey and Terken, 2017), with some arguing for the vehicle’s behavior being more intuitive than the dedicated interface (e.g., Moore et al., 2019). Despite the lack of definitive answer as to the effect of those systems, eHMIs could be one of the ways of increasing the trust in and acceptance of highly automated vehicles, especially in times of transition from manual to full vehicle automation.

Within those who do find value in the eHMIs as a way of communication with other road users, there is no consensus, though, as to the specifics of that communication (e.g., Faas et al., 2020; Mahadevan et al., 2018; De Clercq et al., 2019; Ackermann et al., 2019; Merat et al., 2018). Literature surveys which analyzed and categorized existing concepts (Löcken et al., 2019; Colley et al., 2020; Dey et al., 2020a; Schieben et al., 2019; Mahadevan et al., 2018) found the textual and symbolic communication as the most common due to its ability to convey more complex messages. Dey et al. (2020b) proposed well-established red for “stop” and green for “go” and more neutral cyan for yielding could be used for communication *via* light band eHMI, and U e was used for an often indecisive research field. The SAE Recommended Practice J3134 (SAE, 2019) advises using two symmetrical, continuously lit blue-green light signals as a way of communicating an automated state.

The true value of eHMIs for automated vehicles has yet to be determined. Still, both for traditional and automated public road transportation means, a number of issues and application areas related to a lack of communication have been identified. Rapid acceleration and harsh braking while arriving at or leaving a bus stop, for example, often lead to injuries of passenger waiting, boarding, off-boarding, as well as on board on the bus or shuttle (Wretstrand et al., 2014). Apart of being potentially dangerous for the passengers, docking the vehicle into a bus stop is also a stressful moment for the driver who needs to both perform the maneuver, as well as to communicate with waiting, boarding, off-boarding, and on-board passengers.

There is evidence suggesting that the automation of the docking procedure could increase safety and lower drivers’ stress levels (Ahlström et al., 2018). Tests in contexts ranging from dedicated lanes of Bus Rapid Transit (BRT), test tracks, and open urban roads, further showed improved precision (Collet et al., 2003; Huang and Tan, 2016; Tan et al., 2002; Yoshioka et al., 2014), improved ease of access and waiting time for all passengers, and loading and unloading time for those with special needs (Huang and Tan, 2016).

A study by Collet et al. (2003) indicates that automated docking, after initial habituation, reduced drivers workload as they no longer have to maneuver the vehicle but only monitor the process. A similar decrease in workload would not be observed when the drivers were unfamiliar with the system or faced with an error or takeover. eHMIs can be a solution in such situations of a higher communication need by providing additional, universally understandable, and always-available communication means, which can support not only the drivers but also the passengers in automated traffic transportation environments, especially in situations of risk and conflict.

Due to the challenging nature of designing and assessing eHMIs in real traffic, research in this particular area is still rather scarce (Colley and Rukzio, 2020; Dey et al., 2020a). Most eHMI research has been conducted in, for example, Colley et al. (2020); Dey et al. (2021); Faas et al. (2021); and Mahadevan et al. (2018) and about various forms of simulated traffic so far (e.g., Hoggenmüller et al., 2021), but studies in real traffic contexts are still scarce.

## 3 Studies overview

The starting point for this publication was the work that had been conducted throughout the Austrian national flagship project Digibus® Austria. The general aim of this project was to investigate the integration of fully automated shuttles into the existing traffic infrastructure as a last-mile solution. Working on eHMI solutions for communication with pedestrians and other road users was only one of several facets within the project. Along the project, initial user requirements were gathered, and eHMI solutions were proposed and conceptually evaluated for a variety of interaction contexts and multiple types of road users. As things usually go in these kinds of investigations, only a subset of these solutions could be carried forward for the field tests and were evaluated in some—but not all—of the contexts that had been identified as critical in the initial analysis.

Although this is par for the course for virtually any research activity, we had been in touch with the two other research projects, *auto.Bus—Seestadt* and *Drive2TheFuture*. We found that together the three projects covered a wide and very synergistic area, which, in addition, addressed the initial scope of *Digibus® Austria* quite well. We decided to pool our resources and results together to provide a more comprehensive overview of what worked (and what did not) in terms of eHMIs for external communication of automated shuttles.

**FIGURE 1:**
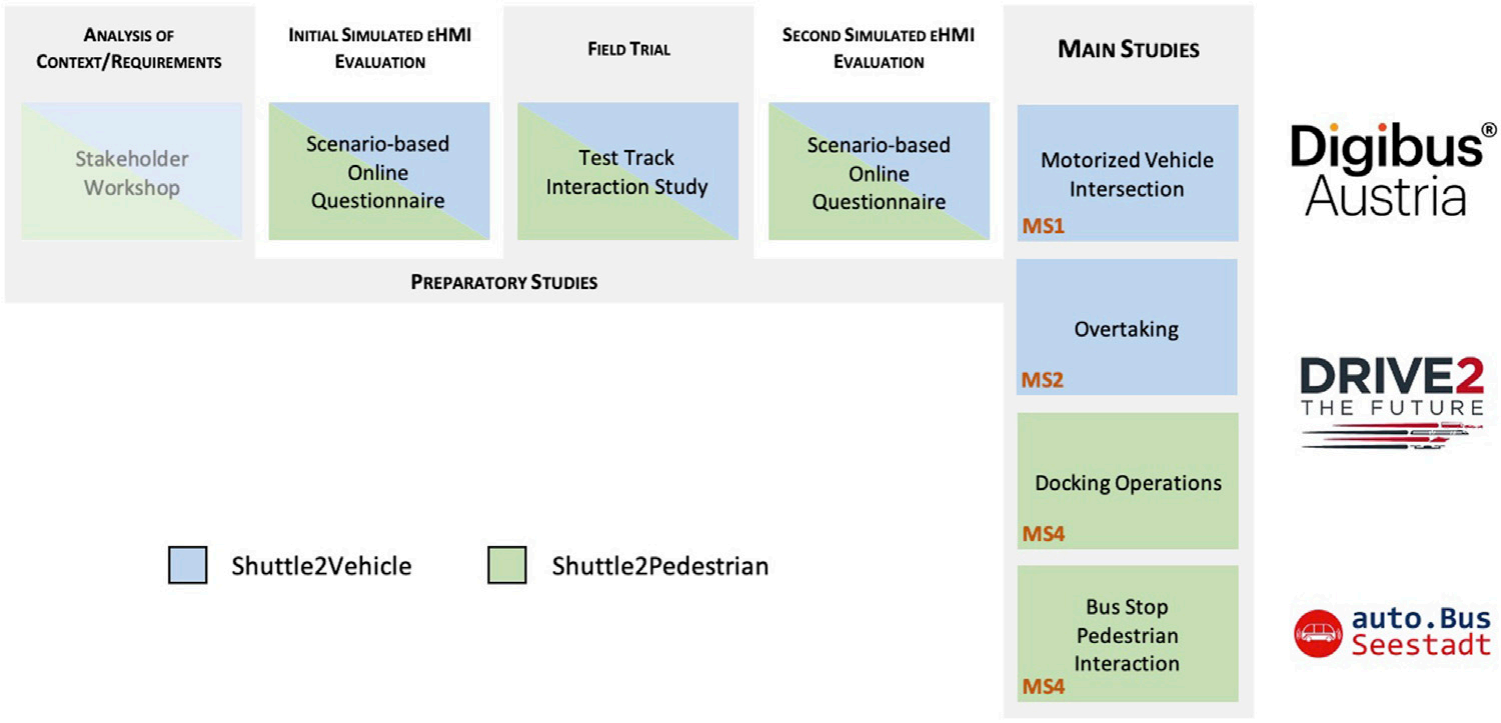
Overview of all studies included in this publication from each participating research project. Note: The stakeholder workshop was the general starting point feeding into all further research activities. For reasons of clarity and scope, its exact procedure will not be discussed further in this study, but the key data and essential findings are outlined as part of Section 4.1, as its essential findings were immediately applied in the first online questionnaire study.

This study describes the preliminary activities and preparatory studies from *Digibus® Austria* and then branches out to describe four main studies from all three participating projects (see Figure 1 for an overview). The preliminary studies identified a number of critical scenarios and provided two online evaluations of eHMI designs to address a wide range of them, along with an initial field trial in-between. The eventual field study (MS1) could cover eHMI designs to communicate with motorized vehicles in crossing situations but none of the other relevant scenarios. A field study conducted in Norway (MS2, *Drive2TheFuture*) investigated an eHMI to reduce dangerous overtaking situations, whereas a co-simulation study in Sweden (MS3, *Drive2TheFuture*) and another field study in Austria (MS4, *auto.Bus—Seestadt*) investigated eHMIs to support pedestrian/passenger communication for bus stop docking and boarding operations. All preliminary studies as well as MS2 and MS4 are original, previously unpublished research. MS1 and MS3 have both been published individually; the full publications are referenced in the respective study sections, and the sections contain abridged summaries of setups and results to convey the essential findings and lessons learned.

## 4 Preparatory work and preliminary studies

Traffic configurations—both in terms of the physical environment as well as traffic participants—are manifold. Thus, the first step consisted in identifying the most relevant of these traffic configurations and then focuses the research efforts accordingly. To this end, one of the first activities in the *Digibus® Austria* project was an expert workshop in October 2018, where these configurations were identified and captured as concrete interaction scenarios. For each of these, a number of eHMI concepts were developed for automation-critical situations (i.e., situations, where there was an additional challenge due to the shuttle’s automated nature and not only it being difficult to handle traffic situation in general). These were then conceptually evaluated *via* an online questionnaire in April 2019. Based on the lessons learned from that study, a second iteration of the eHMIs was created and deployed in the first field trial in November 2019 and then evaluated in a second online questionnaire in March–May 2020. These three activities constitute the preparatory studies, which we will report in the following. A selection of the eHMIs was then realized for the eventual main field study, which will be reported in Section 5.

### 4.1 Interaction scenarios and preparatory eHMI designs

The first preparatory study was a scenario-based, online evaluation of three different eHMIs that were designed to support an automated shuttle in communicating its driving intentions to other groups of road users in potentially ambiguous traffic scenarios. The traffic scenarios that the eHMIs would address were chosen based on an expert stakeholder workshop. The workshop was a half-day workshop, and the experts were from a variety of different backgrounds; from representatives of the public transport sector to urban transport planners to spokespersons for vulnerable road users. The scenarios, which the experts, finally, agreed on to hold a lot of potential for ambiguous driving and communication behavior to occur, when an automated shuttle and other groups of road users meet in traffic, were the following:

• **Crossing (C):** The shuttle approaches a zebra crossing and communicates that it has recognized the pedestrians, who are about to pass, and that it will stop in front of the zebra crossing accordingly.
• **Unregulated junction (UJ):** The shuttle and another road user (car, motorbike, and bicycle) are approaching an unregulated T-junction at the same time from different directions. From the other road user’s perspective, the shuttle is coming from the right therefore has right of way and, also, communicates that it will pass the intersection and not give up on its right and wait for them to pass.
• **Regulated junction (RJ):** The shuttle and the oncoming traffic have a green light at a regulated junction and are allowed to continue driving straight ahead. However, the shuttle wants to turn left at the crossing, but then it has to abort its turn before it crosses the oncoming lane due to pedestrians crossing in the side street. The shuttle is communicating that it has stopped for the pedestrians in the side street and is not driving any further as long as the way is not clear.
• **Boarding (B):** A prospective passenger approaches the shuttle stop or is already standing in the shuttle stop and wants to know if they can still make it onto the shuttle in time, or are still allowed to board the shuttle. The shuttle communicates that it is time to get on board the shuttle and alerts the passengers in the vicinity that it is about to leave, before it closes its doors.
• **Passing (P):** The shuttle passes an oncoming pedestrian on the side of a road without pavement and communicates that it has recognized the pedestrian and that it is keeping enough distance while passing them, so they can proceed walking unaffected.

For the first questionnaire, these were adapted directly, resulting in eHMI evaluations across five interaction scenarios. For the second questionnaire, the crossing and junction scenarios were further refined into two separate sub-scenarios each (A and B). For the crossing scenarios, A had the shuttle approach the crossing from a distance, whereas B had the shuttle stopped in front of the crossing and attempting to accelerate. Likewise, the junction scenarios were differentiated by the shuttle approaching the junction from a distance (A) or waiting at the junction (B). Passing was dropped, since it had turned out to be difficult to realize during the field trial, whereas Boarding remained a single scenario. This resulted in a different number of scenarios for the second questionnaire (seven in total), although both questionnaire studies are based on and are consistent with the base scenarios described before.

#### 4.1.1 Initial eHMI designs

**FIGURE 2:**
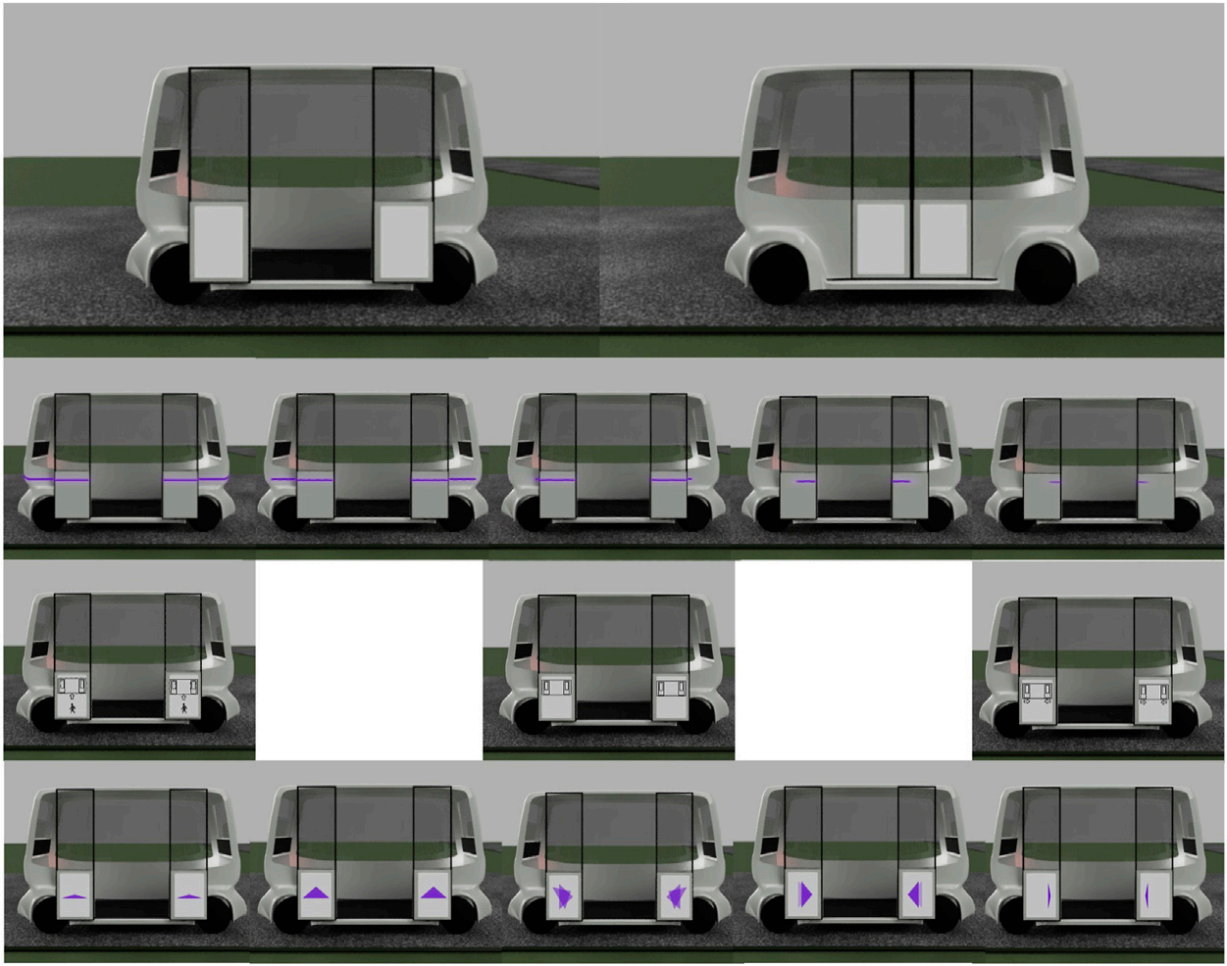
Boarding—overview of all initial designs.

The three eHMI designs (see Figure 2 for a representation of each in a boarding scenario) were developed internally by a team of user experience (UX) and design researchers in a half-day creative workshop, for which the five traffic scenarios provided the parameters for. After a process of freely creating several solutions, ideas were sorted, refined, and iterated, which, finally, led to three general design paradigms, each of which was able to be applied in all five traffic situations. These were as follows:

• **Morphing arrows** that can provide information about trajectory, acceleration, and braking behavior as well as detected objects in the immediate vicinity of the shuttle *via* shape change and rotation.
• **Icons** that can provide information about the traffic environment perceived by the shuttle. In addition, escalation steps can be indicated by changing from a static display to flashing.
• **LED bars**, mounted laterally around the shuttle, that can provide information on the intended driving behavior and, to a limited extent, also on the detected traffic environment by means of light frequency, rhythm, movement, and range.

In order to avoid associations with already existing light signals in road traffic, a light shade of violet was chosen as the uniform color of light signals. Furthermore, in order to counteract potential liability issues, it was defined that the information communicated by the shuttle only addresses currently available sensor information and current or immediately planned driving behavior and intentions, whereas transmissions of instructions or information, which could directly or immediately prompt other road users to take action, were explicitly excluded. These designs were evaluated in the first online study.

#### 4.1.2 Iterated eHMI designs

**FIGURE 3:**
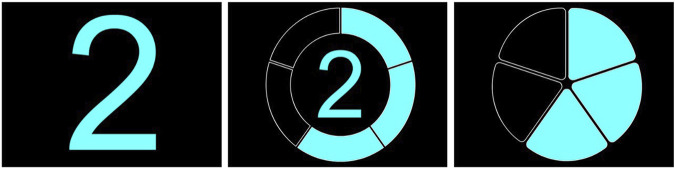
eHMI design countdown as applied for the field study.

**FIGURE 4:**
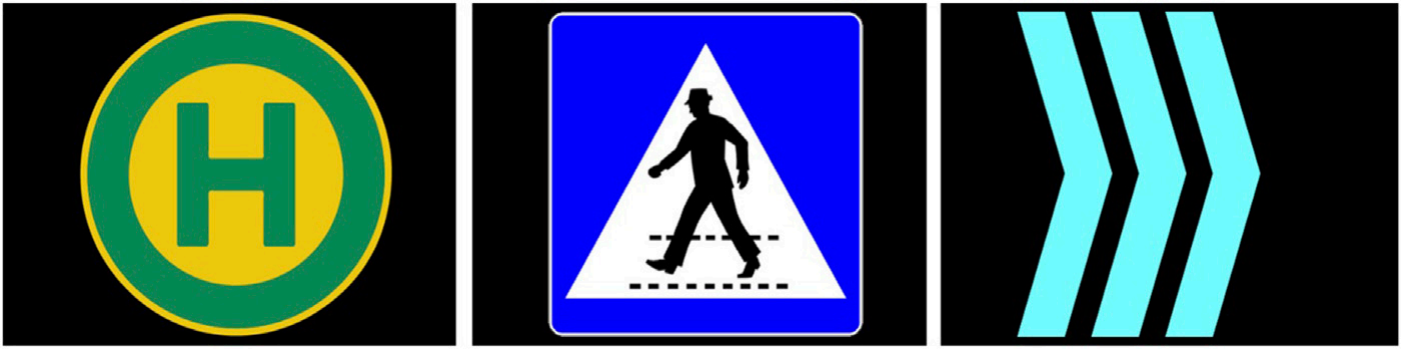
eHMI design icons and arrows as applied for the field study.

**FIGURE 5:**
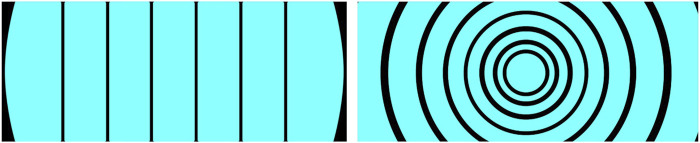
eHMI design animated bars as applied for the field study.

Based on the lessons learned from the first online study and the technical and contextual characteristics of the shuttle and the driving track, an internal workshop was conducted to derive and define the next iterated set of eHMI designs (see Figures 3–5).

• **Countdowns:** Three different designs of animated 5-s countdowns were implemented. The goal with these was to compensate for the shuttle’s extended start interval and clearly communicate the driving initiative (i.e., the exact point in time, when the shuttle would depart). In this regard, they were also intended to be particularly helpful in resolving “deadlock situations” (e.g., at unregulated intersections, where both vehicles are at a standstill). Also, due to the conflicting aspects of readability and visibility over distance, now that people were no longer sitting in front of a screen but observing the shuttle from a far as pedestrians and car drivers, a numbers-only design, a design with numbers embedded in a half pie and a full pie design without any additional numbers, was implemented. The animation sequence for each of the countdown-based eHMI designs consisted of one animation cycle counting down from five to zero or from five empty to five filled segments, followed by a 2-s flashing interval of the final animation for all types of countdowns. So, in total, it took about 7 s for one animated countdown to be fully displayed.
• **Icons and arrow animation:** In alignment with the results from the first online questionnaire, where the icons and animated arrow eHMI designs were received as the most suitable designs to communicate the shuttle’s driving intentions, two standard icons, one for a stop and one for a pedestrian crossings, were implemented. Also, the animated arrow design from the first study was changed to a simpler but easier to perceive version, which should also address the issue of readability and visibility over distance. The design goal was not only to communicate the shuttle’s intentions but also information on the contextual awareness of the shuttle to the car drivers and pedestrians.
• **Animated bars:** As an alternative design to compensate for the extended start interval of the shuttle as well as to possibly gain insights into the level of detail and concreteness necessary to communicate the driving initiative, two additional simplified and animated eHMI designs were realized. They came in two shapes (bars and circles) and at three different speeds. The bar design was mimicking an LED strip, basically, and was an approach to further pursue the communication capacities of an LED-like information design, as the LED bars were not received positively in the first online study. The circle design was more of a creative approach to address the issue of recognizeability through a kind of twitchy, unconventional but attention-seeking design. Both eHMI were animated to expand or contract to or from the center depending on the chosen direction (in-out or out-in) at speeds of 3, 4, or 5 s. An additional design goal was to communicate the planned start or planned stop of the shuttle in advance (i.e., before any acceleration or deceleration is apparent from the driving behavior) to make the shuttle’s driving intentions even clearer to car drivers and pedestrians.

These designs were used for both the initial field trial as well as the second online study. The only exception was the animated circle design (see Figure 5). Since there were no meaningful advantages and only disadvantages (distraction and confusion) reported during the initial field trial, it was not carried forward afterward and only the straight bar design remained.

### 4.2 Study setups and methods

The main research goals of all three preparatory studies was to find out whether or to what extent the eHMIs could help other road users better anticipate the shuttle’s driving intentions. The guiding research questions (RQ) were as follows:

• RQ1: Are the eHMIs recognized by other road users as relevant to them?
• RQ2: Are the eHMIs successful in communicating the intended content?
• RQ3: How suitable are the eHMIs for resolving, due to the lack of a human driver, potentially ambiguous traffic scenarios?

#### 4.2.1 Online questionnaires

**FIGURE 6:**
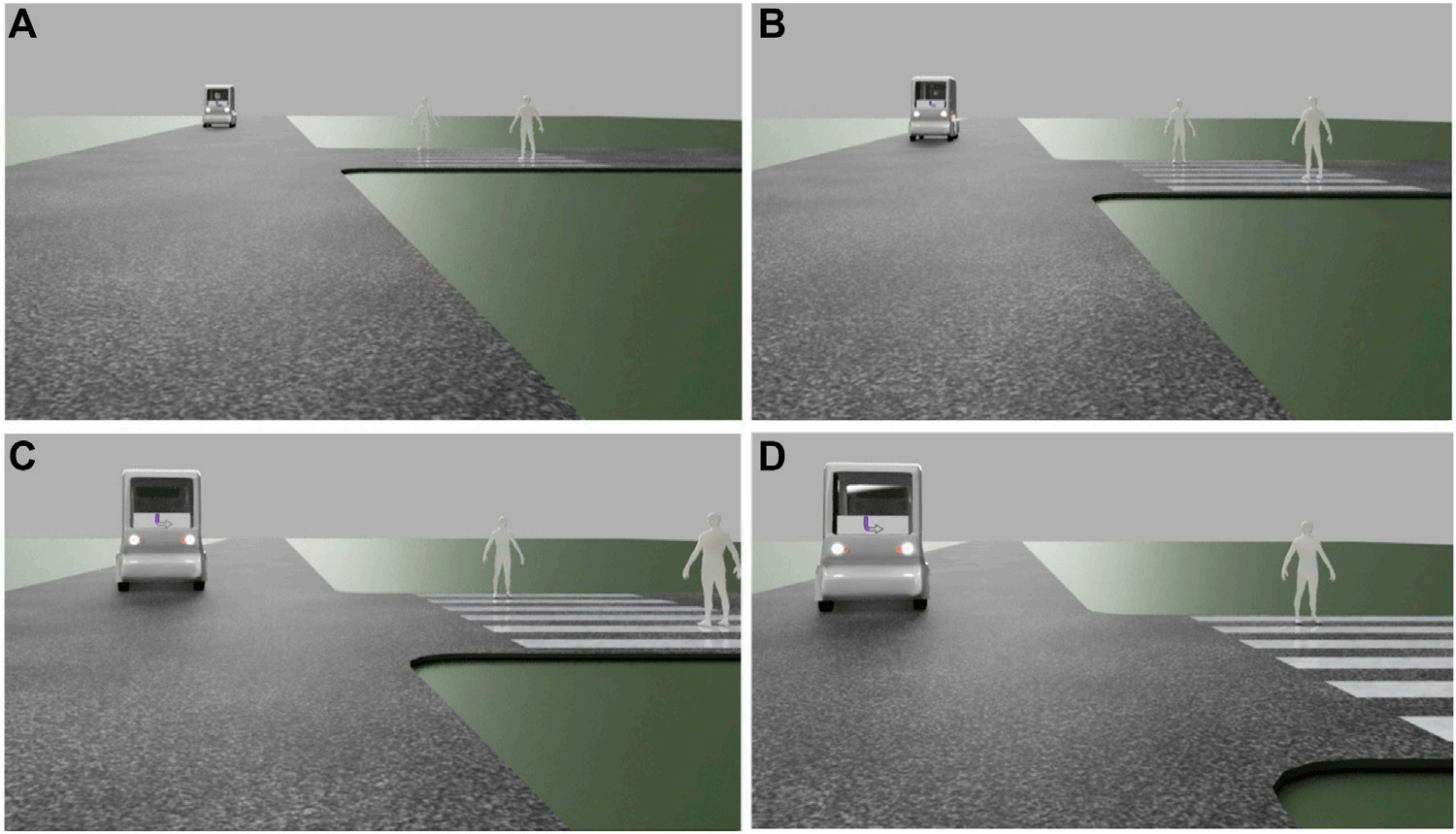
Visualization example [animation stills, sequence from **(A–D)**] of how the scenarios were presented in questionnaire 1.

**FIGURE 7:**
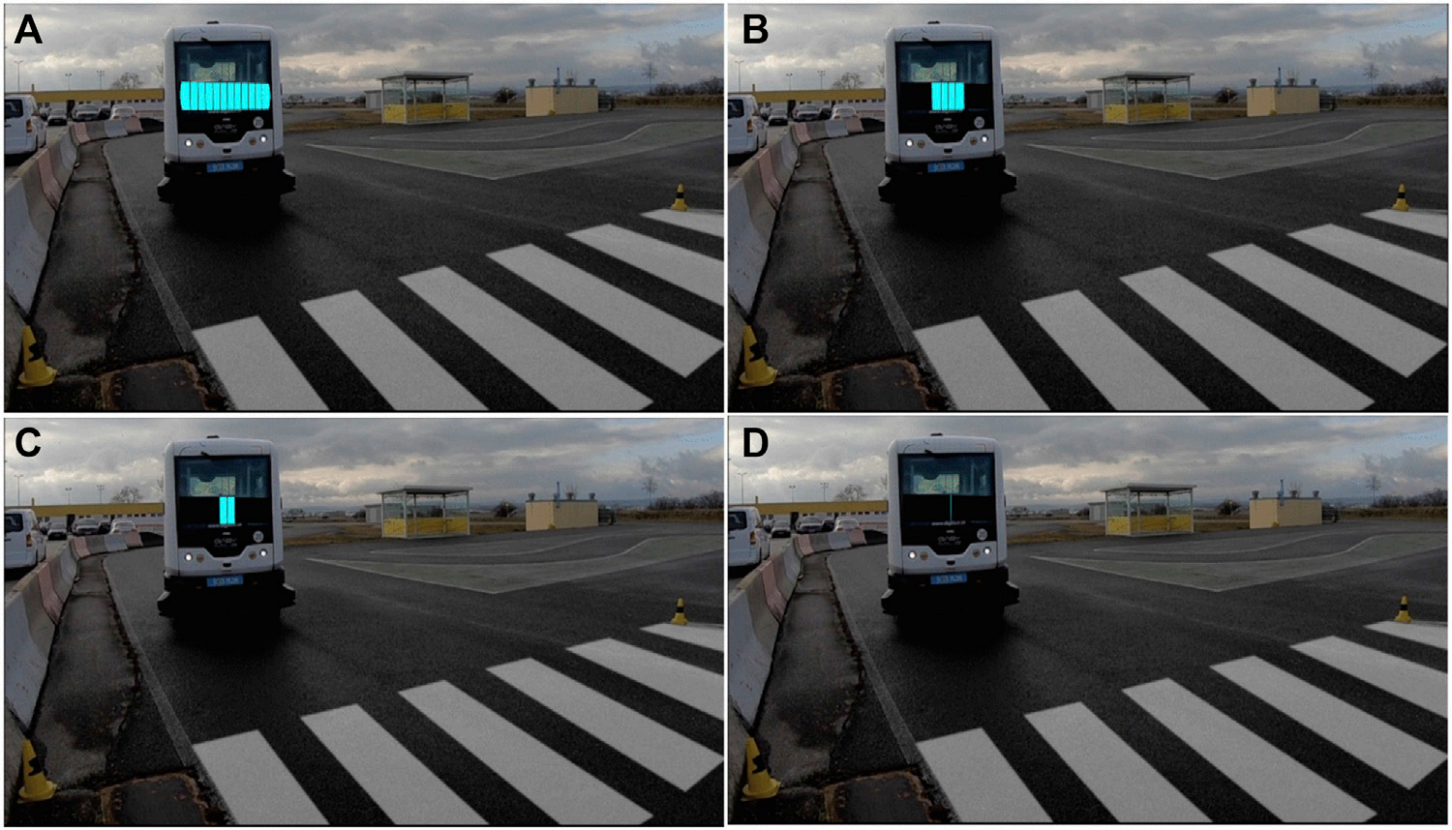
Visualization example [animation stills, sequence from **(A–D)**] of how the scenarios were presented in questionnaire 2.

The traffic scenarios were first drawn as storyboards with a short text description from the first-person perspective of a road user approaching the shuttle for use in the online evaluation. For the first online questionnaire, the scenarios were implemented as 3D animations, based on a simplified model of the shuttle (the template of which we had received from the shuttle manufacturer, Navya). The scenarios were created in Blender and then embedded in Photoshop. The three initial eHMI designs (LED bars, morphing arrows, and icons) were implemented as part of the shuttle’s eHMI for each traffic scenario (see Figure 6). The second questionnaire used videos from the actual shuttle on the test track, overlaid with the iterated animations (see Figure 7).

The online questionnaires were both set up in LimeSurvey and followed the same structure and format. The interaction scenarios were realized as animated gif images and integrated into the survey to constantly loop so that participants would be able to watch every scenario several times without missing anything. Before the scenarios were introduced, participants were asked for some demographic data (age, gender, driving license possession, frequency and mode of transportation usage, and experience with automated vehicles). Then they were presented with the scenarios in a random order.

For each condition in each scenario, participants had to give their opinion on the following statements to evaluate their interpretation of the situation and the shuttle’s driving intentions:

• **safety:** I feel safe to continue my journey.
• **action2:** It is clear to me that the shuttle is going to turn left in front of me.
• **perception1:** It is clear to me that the shuttle has noticed me.

Furthermore, to evaluate participants’ opinion on the general recognizability and interpretability of the eHMI at display, they were asked to rate four more statements:

• **visualization1:** I would assume that most people would quickly recognize what the visualization means. (*SUS7*)
• **visualization2:** I find the visualization unnecessarily complex. (*SUS2*)
• **visualization3:** I understand the meaning of the visualization straight away. (*SUS10*)
• **visualization4:** I find the visualization superfluous.

Finally, to learn something about participants’ preferences regarding the eHMIs per scenario, participants were asked which visualization was the best to detect the shuttle’s driving intentions. The statements on safety, action2, and perception1 were adapted in phrasing to fit the respective scenario at hand (e.g., for the boarding scenario, the statement on safety read like this: *I feel safe to be able to get on the shuttle in time.*) The first three statements on visualization (1–3) were taken from the System Usability Scale (SUS). For the control condition, which was not displaying any visualizations, only the statements safety, action2, and perception1 were used. For all statements, answers ranged from *not at all (1)* to *absolutely (5)* on a five-point Likert scale. For the question for the preferred eHMI, the selection was single-choice with an additional *none of the shown* option.

The first questionnaire evaluated four conditions (the three initial eHMI designs and a control condition without designs) across the five defined interaction scenarios, and the second one across seven scenarios (see Section 4.1).

#### 4.2.2 Initial field trial

**FIGURE 8:**
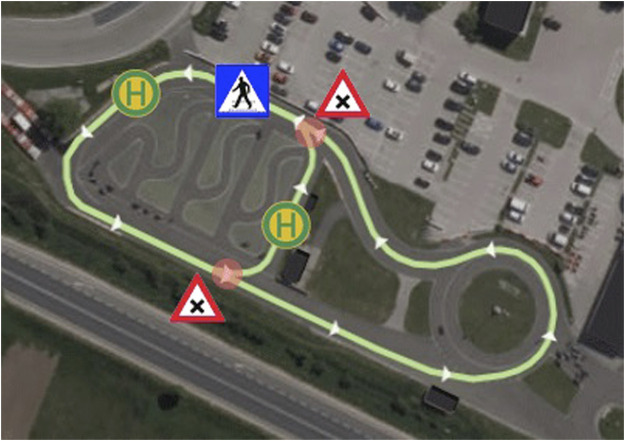
Top-down view of the test track showing the pedestrian crossing, vehicle intersections, and bus stops.

The initial field trial was conducted on a test track at a driver training center (see Figure 8), where the actual shuttle equipped with the different eHMI, car drivers, and pedestrians interacted with each other under semi-realistic traffic conditions. The field trial was conducted after the first online questionnaire and used the iterated eHMI designs described earlier. The main objective of the study was the *in situ* evaluation of the iterated eHMI designs. To this end, RQs 1–3 were extended with the following evaluation targets:

• **Visibility/recognisability:** How well are the designs recognizable to the other road users (pedestrians, car drivers)? Do they attract their attention or not?
• **Directedness:** Do the other road users feel addressed?
• **Comprehensibility:** How do the other road users interpret the meaning of the designs?
• **Usefulness:** How useful do the other road users experience the designs?
• **Trust:** How much do the other road users trust the behavior of the bus based on the designs?
• **Subjective safety:** How safe/unsafe do the other road users feel in the respective situation? Are the designs perceived as promoting safety?
• **Alternative design suggestions:** Do the other road users have alternative suggestions for what the designs could look like?

Quantitative data was collected along these factors *via* a post-interaction questionnaire, with one item for each of the initial six (visibility–subjective safety) to be answered on a 5-point Likert scale. The items asked were closely related to the research questions and the wording was adapted to fit pedestrian as well as car driver scenarios. An observation log was used to record contextual information such as the date, time, duration, weather, and group size (for pedestrian and passenger groups), and whether or not the currently displayed eHMI in the respective situation led to a successful completion of the scenario.

In a final interview, the participants were asked which of the eHMI designs they had consciously noticed and for their interpretation of the information provided. Additional questions concerned the perceived eHMI addressees, whether the eHMI was perceived as helpful or unnecessary, whether the eHMI contributed to perceived safety, and suggestions for improvement. Basic demographic data (age, gender, place of residence, information on visual or hearing impairments, and previous experiences with automated vehicles) was collected as well.

### 4.3 Preparatory studies results

In the following, the results from the questionnaires and initial field study are reported. All analyses were conducted using R, SPSS, and Excel unless indicated otherwise. The questionnaire results are limited to which design was preferred by the participants per scenario. The complete results regarding the performance of each design within each individual condition from both online questionnaires are reported in the Supplementary Material. Due to the low N and procedural difficulties (explained in more detail below), the field study results are focused on the qualitative data.

#### 4.3.1 Online questionnaire 1 results

The first online questionnaire was answered fully by 83 individuals (mean age 45 years (SD = 19.5): 52 men (62.7%) and 30 women (36.1%) (missing = 1). Almost all participants (97.6%, N = 81) stated that they had a driving license. About half of the respondents (53%) stated that they had never been traveling with a self-driving vehicle, while 47% had already experienced riding in one before (subways 51%, automated buses 19%, trains 15%, and cars 13%).

**FIGURE 9:**
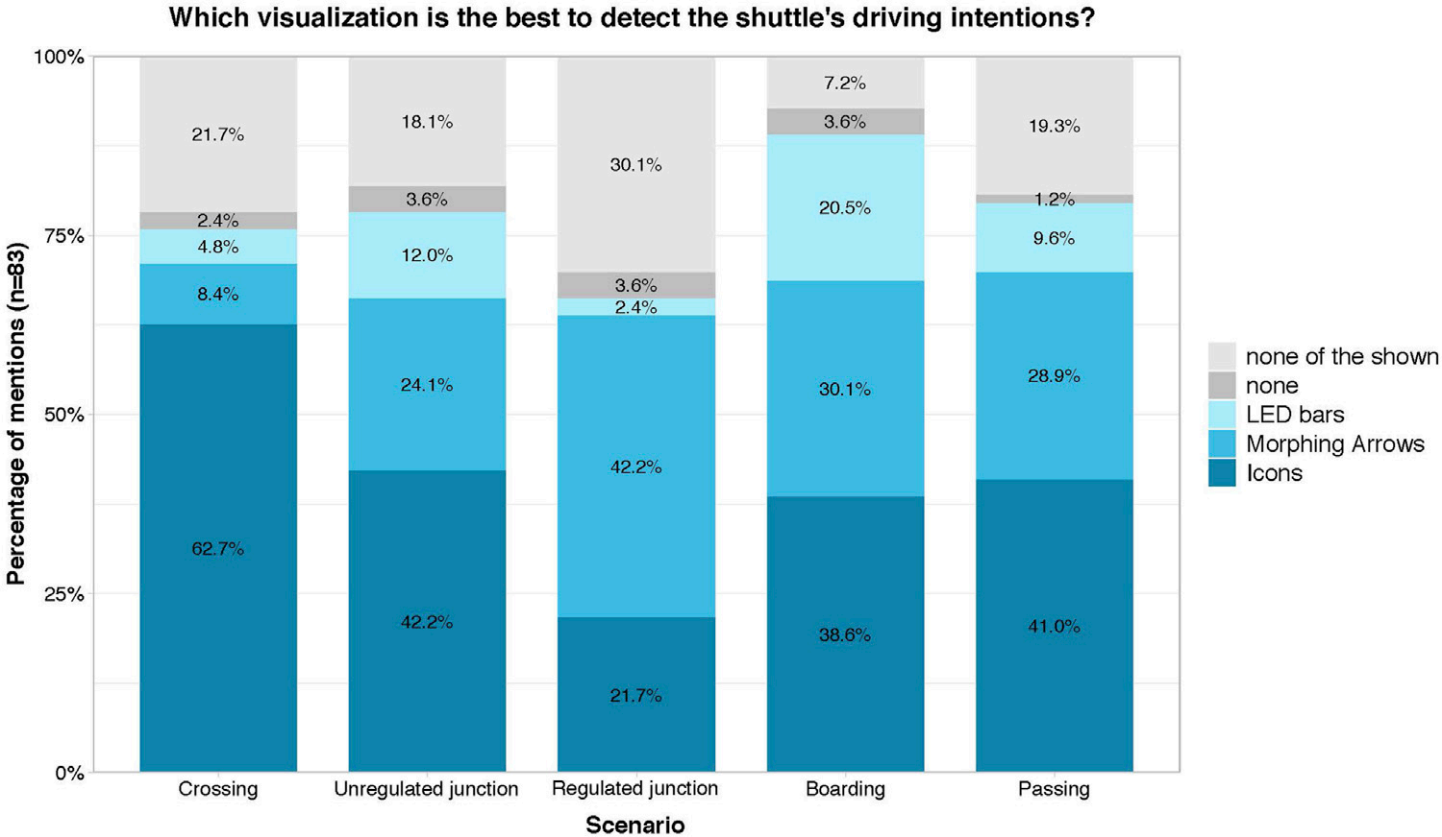
Preferred eHMI for detecting the shuttle’s intentions.

In general, the participants preferred the icon-based eHMI most frequently in all scenarios except for the regulated junction scenario, (see Figure 9). At the same time, the respondents generally seemed to find an eHMI necessary in order to recognize and understand what the automated shuttle is about to do. The proportion of people who stated that they can best recognize the shuttle’s intention without any design was very low in all scenarios (between 1.2% and 3.6%).

In the scenarios crossing, boarding, and passing, the icon design is chosen by about 40% of the people, followed by the arrows design with about 25–30%. The LED visualization is chosen significantly less often in all scenarios. Only in the boarding scenario is the proportion higher, at 20.5%. It is also noticeable that the distribution of the answers to the different eHMI designs is most balanced in this scenario. This is also consistent with the individual evaluations, in which the designs perform very similarly for boarding. At the same time, in this scenario only 7.2% of the respondents indicate, that they did not find any of the variants shown to be the best.

In contrast, in the regulated junction scenario, the proportion of those who are not satisfied with any of the variants shown is significantly higher (30.1%), and the highest compared to all other scenarios. In this scenario, the icon design is also chosen less often (21.7%) and the morphing arrows design is preferred more strongly instead (42.2%). One reason for this is that, in comparison, the arrows eHMI was rated more often to be rather comprehensible and immediately understandable for many people, while the icons eHMI was rated as unnecessarily complex and at the same time not very comprehensible. In general, however, none of the presented eHMI seemed to be able to convey an adequate sense of safety or a sufficient level of information of what the shuttle is up to in the regulated junction scenario. Ratings regarding these aspects are generally lower in this scenario.

Overall, participants preferred at least one of the eHMI designs over having none eHMI at all in each of the presented traffic scenarios. However, the results were inconclusive with respect to RQ1 and RQ2 (are eHMIs recognized by road users and do they communicate the intended content?) and also varied greatly depending on the individual eHMI and the respective traffic scenario. For the regulated junction scenario in particular, no eHMI was perceived as overly successful in communicating the intended information. Regarding RQ3 (Can the eHMI resolve ambiguous traffic situations?), the icons-based eHMI was received as most suitable, for all but one traffic scenario, the regulated junction, where the morphing arrows performed best.

Communication *via* the LED bars turned out to be surprisingly ineffective across all scenarios and resulted in it being the least preferred of the three designs. For the following implementation and field tests, a combined solution of improved icons- and arrow-based eHMI was decided as the most logical next step in the development process, with LEDs being excluded for the same reason.

#### 4.3.2 Initial field trial results

A total of 14 participants participated in the field trial: four car drivers and ten pedestrians. Among the participants were nine men and five women. The two youngest subjects were two children aged nine, the oldest participant was 70 years old. The average age was 42. The majority (10 out of 14 people) had not had any experience with automated vehicles before.

Conducting the study was difficult due to a variety of factors, including highly volatile weather conditions during the testing period, presence of other lights and indicators on the shuttle that could not be deactivated (nor could the shuttle’s signaling behavior be controlled directly) and issues with properly simulating the interaction on the test track due to the participants’ often non-realistic interaction with the shuttle due to curiosity (reported separately in Mirnig et al., 2020).

Overall, according to the car drivers, the external communication means of the shuttle were well recognized. But this result has to be interpreted with caution, as it became apparent from the interviews that the participants most probably most of the time referred to the totality of external communication means including the shuttle’s indicator and hazard warning lights and not only the displayed eHMI. The standard behavior of the shuttle during soft stops, which led by default to an activation of the hazard warning lights, contributed to participants’ ambiguous perception of the communication means.

The crossing scenarios in which the bus was in motion were in most cases manageable without any further assistance by additional eHMI due to the study design and the low level of risk. Interestingly, however, the assessments of the predictability of the shuttle’s behavior was rated very negatively. Therefore, a general need for further information but no communication success of the implemented designs can be deduced.

Only one person gave way to the bus in the T-junction scenario, although the car drivers would have been expected to let the shuttle enter the intersection first. It can be concluded that the information the eHMI provided was either not considered as relevant or was not even properly perceived to begin with. Countdowns half-pie + numbers and full pie turned out to be less understandable than numbers only, with the countdowns being, generally, not being perceived fully or just ignored.

For the pedestrian scenarios, however, the countdowns turned out to be more useful, as in all but the countdown conditions, the shuttle was not able to depart as scheduled or had to stop again after having already initiated the start-up. This was, also, due participants’ curiosity and novelty effects, which obstructed compliant participant behavior (see Mirnig et al., 2020).

Also, in the boarding scenario, participants experienced some uncertainty with respect to getting on board in time. Although the eHMI designs were largely not perceived as superfluous, it was not possible to identify one design as clearly more successful and helpful than the others. An overall advantage of the different eHMI designs over the control condition was still noticeable, though, and, also, confirmed in the qualitative results.

Although the sample size was not sufficient for a meaningful quantitative data analysis, the interviews quickly revealed that, especially for the shuttle-to-vehicle interaction designs, a larger sample size would not have changed much, as the issues lay on a more fundamental level. Visibility of the eHMI was limited and would decrease as the vision angle increased. In addition, the reality of traffic interaction meant that the participants’ gazes wandered constantly and did rarely remain on the displays for long enough to see the full animation, which was especially true for the countdown, circle, and bar animations. As a result, overall comprehensibility was low, regardless of how well the animations might have worked in isolation, as they were simply not seen in their entirety most of the time. The eHMIs fared better in the pedestrian interaction scenarios overall but the low number of participants still meant that validation from a quantitative standpoint was not possible.

Due to the high amount of effort required to conduct the study and the number of difficulties encountered, the field trial was ended prematurely after the 14 participants. Since the obtained results were primarily qualitative, it was decided to conduct the second online evaluation to provide the said quantitative validation.

#### 4.3.3 Online questionnaire 2 results

A total of 112 completed responses were recorded. The youngest participant in the sample was 16, the oldest 82 years old (M = 44.37 years; SD = 21.11 years). The gender distribution was almost equal with 55 men and 56 women (one person preferred not to answer). A majority (73%) of the participants came from Austria, another 9% from Germany. The remaining mentions (with an N between 1 and max. 3) were from Belgium, Colombia, Denmark, Estonia, France, Latvia, the Netherlands, Norway, Portugal, Slovenia, Spain, and Sweden. Only 7 participants did not have a driving license at the time the study was conducted. 40% had prior experience with automated vehicles.

##### 4.3.3.1 Pedestrian crossing scenarios

**FIGURE 10:**
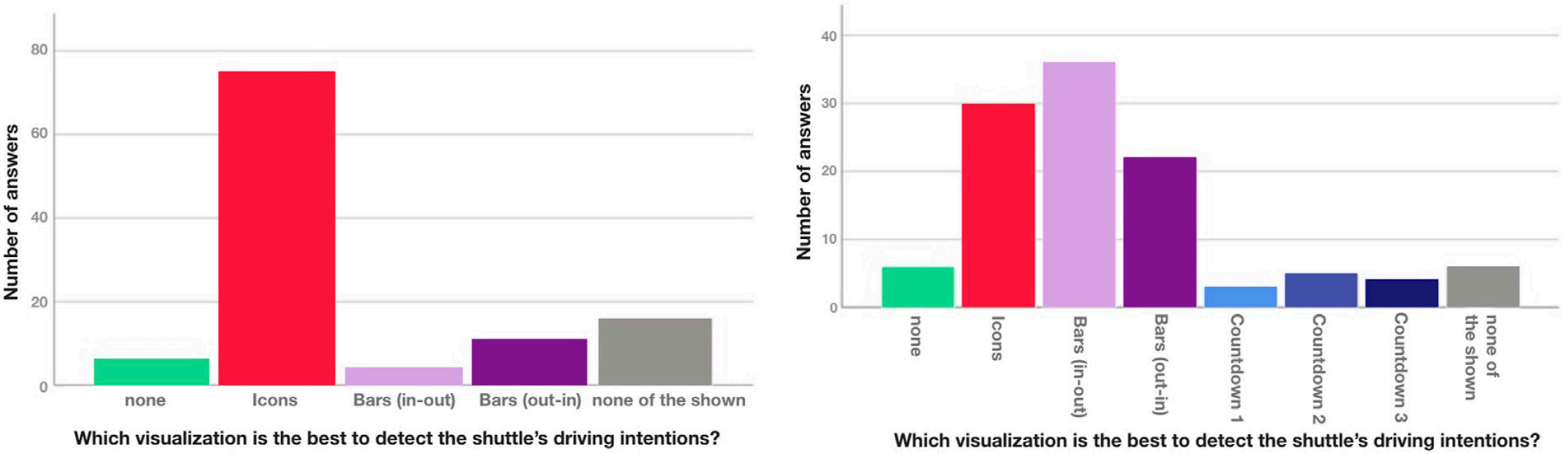
Preferred eHMI for detecting the shuttle’s intentions at Crossing A and B.

In the Crossing A scenario most participants chose the icon-based eHMI as their preferred design (see Figure 10). While the percentage of people who indicated that they could best discern the shuttle’s intent without design was very low in both A and B, it was still higher than *Bar (in-out)* in Crossing A and higher than all three *Countdowns* in B. This means that none of these can be considered viable for either scenario. Both bars performed considerably better in B, with *Bar (in-out)* being rated highest of all visualizations, followed by *Icons*, then *Bar (out-in)*. Overall, *Icons* appear to be sufficient to communicate when the shuttle approaches (A) whereas *Bar (in-out)* is best suited for communicating the shuttles acceleration intention (B), although *Icons* would be decently to well-suited here as well. While it was not terribly high, the number of preferences for *none of the shown* suggest investigating further alternatives, especially for A.

**FIGURE 11:**
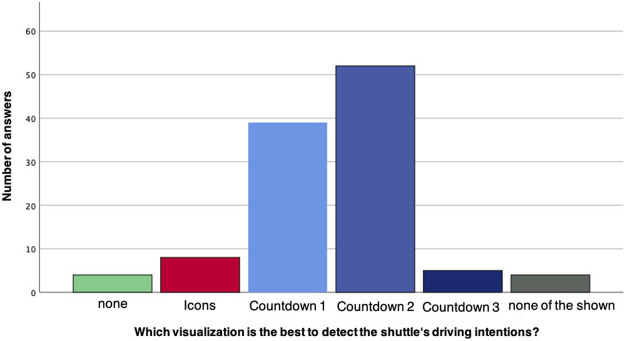
Preferred eHMI for detecting the shuttle’s intentions during Boarding.

For the boarding scenario (see Figure 11), *Countdown 2* (numbers+pie) turned out to be the most suitable one, followed by *Countdown 1* (numbers only). *Countdown 3* (full pie) was rated surprisingly low and *Icons* only slightly higher. Both *none* and *none of the shown* received very low ratings, confirming a need for an eHMI for boarding and *Countdown 2* as a very viable solution.

##### 4.3.3.2 Vehicle interaction scenarios

**FIGURE 12:**
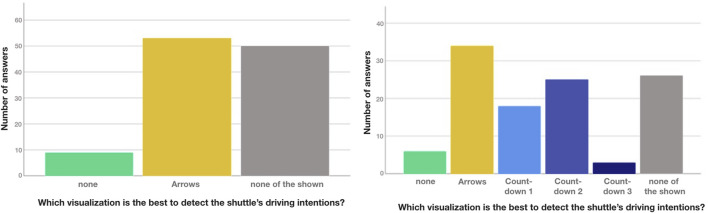
Preferred eHMI for detect shuttle’s intentions at T-junction 1A and 1B

For both T-junction scenarios (see Figure 12), *Arrows* were the highest rated design and the low number of ratings for *none* shows that the participants did feel a need for additional visualizations. However, in both scenarios *none of the shown* was rated very high—almost as high as *Arrows* in A and the highest in B, implying that *Arrows* is far from being the best possible solution. For B in particular, it was assumed that countdowns might be preferred due to giving a clearer indication as to when the shuttle will depart but *Countdown 1*–*3* all performed worse than even *Arrows*, with 1 and 2 receiving decent ratings and three being rated particularly low.

**FIGURE 13:**
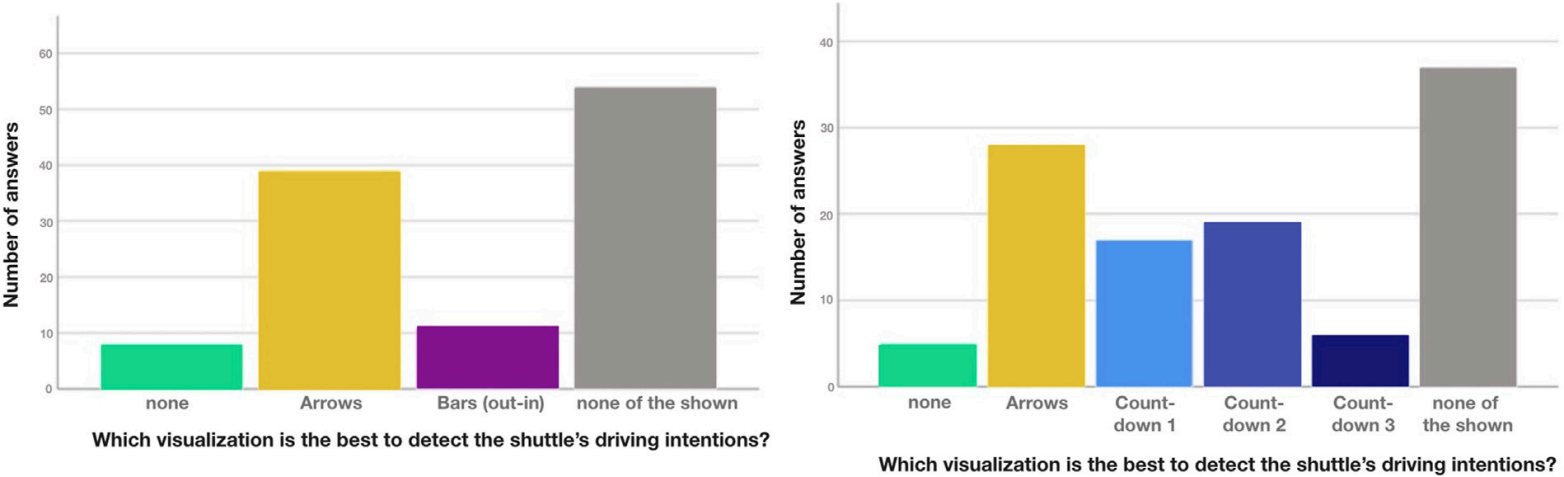
Preferred eHMI for detect shuttle’s intentions at Unregulated junction 2A and 2B

The unregulated junctions were rated similarly (see Figure 13): While in A, the viewing angle (front) allowed use of the frontal display (*Bar out-in*), this condition was only rated slightly higher than *none*. *Arrows* was again the highest rated design, with *none of the shown being rated highest*, suggesting *Arrows* to be viable but not optimal. Unregulated junction B was almost identical to T-junction B: *Arrows* was the highest rated design, followed by *Countdown 1*–*2*, with *Countdown 3* and *none* being the lowest. *None of the shown* was again the highest rated option.

### 4.4 Results summary

The results showed a rather clear interaction path for the pedestrian scenarios: *Icons* work well for communicating the shuttles intent when approaching a crossing and can also be used to communicate its intention to depart, with an animation that shows its intention to accelerate (*Bars in-out*) bringing additional benefit. For boarding, numeric countdowns seemed to work very well, with a visually supported numeric countdown (*Countdown 2*) being the most successful. Interestingly, the abstract countdown design *Countdown 3* (full pie) was rated very low, implying that any countdown design should always have a numerical component in order to be suitable for this scenario.

The vehicle interaction results were less positive regarding the eHMI designs. While the arrows were moderately successful, the high ratings for *none of the shown* across all four vehicle interaction scenarios shows that they might work but are not optimal. Since the participants who answered the questionnaire were not under the time constraints that the participants in the field study had been, it can be concluded that the chosen designs were generally not suitable and that a different approach to the external communication was needed.

The lessons learned during the field study and the questionnaire results pointed toward two main issues: length and visual complexity of the eHMI designs. A suitable eHMI for shuttle-to-vehicle communication would need to be visible in its entirety in as few glances as possible (ideally one) and easily comprehensible in order to not increase cognitive load. Thus, the decision was made to revisit the one-dimensional LED stripes from the first questionnaire study, despite their rather poor performance there. The low resolution and limitation to one dimension would prohibit complex designs and light signals would be easier to see from multiple angles. The original goal had been to realize a comprehensive eHMI with displays for pedestrian interaction and LEDs for vehicle interaction, but only the latter would be realized for the resulting main study for several reasons, primarily the COVID-19 pandemic, which caused few to no pedestrians being on the road during that time.

## 5 Main studies

In the following, the four main studies (MS1–4) from the participating projects are reported. MS1 (conducted in late 2020 and early 2021) was a direct follow-up from the preparatory studies and investigated eHMI designs for resolving encounters with other vehicles in a joint study between *Digibus® Austria* and *auto.bus Seestadt*. MS2 (2020) investigated an eHMI for bicycle overtaking (very similar to the initial *passing*-scenario) in a field as part of the *Drive2theFuture* project. MS3 (2021) investigated passenger docking (entering and exiting the shuttle) scenarios *via* VR co-simulation and was also part of *Drive2theFuture*. Since the designs used LEDs, these well supplemented the ones used in MS1. MS4, a field study from *auto.bus Seestadt* also conducted in 2021, finally adds insights on docking and passenger turnover with eHMIs on not only the shuttle but the station and a wearable as well, thus rounding off the studies both from a methodical and an interface perspective.

### 5.1 Salzburg and Vienna, Austria—Shuttle2vehicle communication (MS1)

The field study that directly followed the preparatory studies, focused primarily on interactions with other road users in situations with crossing trajectories (intersections). This decision was made because the performance within these situations using the previously proposed indicators had still been the most unclear in the preceding questionnaire studies. At the same time, a focus on interaction with motorized road users was also considered to investigate a more unique aspect of eHMI-based communication, as opposed to, for example, boarding situations. At the time of the field study (Nov–December 2020, February 2021), the COVID-19 pandemic was at a high with even a soft lockdown being in effect during (the study days in 2020). This resulted in the scope having to be trimmed down further to only investigated encounters with motorized road users, as the number of pedestrians or cyclists on the road during that period was minimal. The results from the field study were published in Mirnig et al. (2021). In this study, we briefly outline the setup and highlight the most relevant results. For the full study report, please refer to the original publication.

**FIGURE 14:**
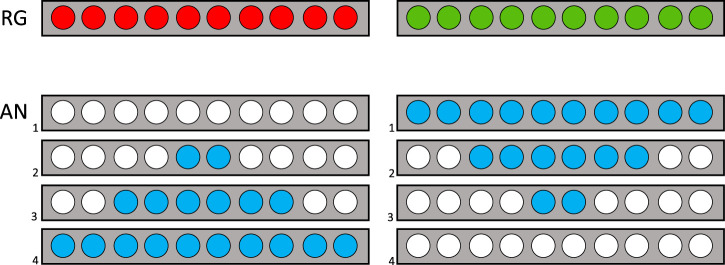
Overview of the two conditions Red-Green (RG) and Animation (AN).

Two designs were realized for this field trial, both *via* a front-mounted LED strip below the windshield, spanning the entire front of the shuttle and bending across to cover the areas front-right and front-left as well, so that the visualizations could be seen even when not being directly in front of the vehicle. The first design *RG* (Red-Green) used a simple traffic light metaphor to signal that the shuttle would either decelerate with the intention to yield (green) or accelerate with the intention to take precedence (red). The second design, *AN* (Animation) communicated the same information but used animations of the LED-bar filling (shuttle accelerates) or emptying (shuttle decelerates) in a neutral light blue color instead. See also Figure 14 for an overview of the conditions.

**FIGURE 15:**
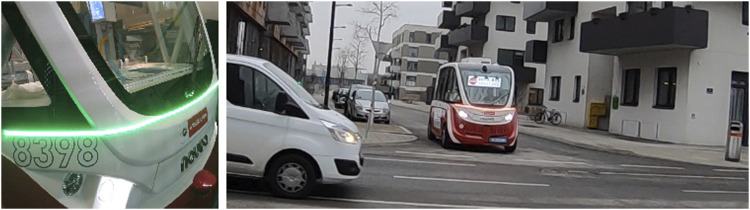
Front-mounted LED on the Navya Arma on close-up (left) and while the shuttle is circulating on the Vienna track (right).

The study was conducted as a joint study between *Digibus® Austria*, *Drive2TheFuture* and *auto.bus Seestadt*. Each project equipped one shuttle with a frontal light band eHMI and deployed it with both conditions (plus a control condition, where the strip was off) on their respective public testing environment (the rural town Koppl bei Salzburg and urban environment Seestadt Aspern in Vienna). See Figure 15 for an example of the Shuttle on the track in Vienna during a drive in the RG condition. Both tracks featured a number of intersections with potentials for crossing vehicle trajectories. The data collection was performed *via* observation and logging, whether conflicts occurred and how situations where the shuttle encountered other vehicles were resolved. For each possible interaction (crossing other vehicles at intersections or joining roads) success and failure conditions were defined [who yields to whom, does an initiated maneuver have to be interrupted (e.g., sudden braking), etc.]. The conditions were then compared by the numbers of successes and failures across all situations.

The results showed an overall success of the eHMIs. There was an assumption that especially *RG* could be confusing, since the traffic lights could be understood in the standard sense (red: other vehicles need to stop) or in relation to the vehicle (red: shuttle stops, other vehicles can go). This assumption was not confirmed, however, as there were no significantly increased communication failure rates by *RG*. However, while both *RG* and *AN* performed well overall, the success rate was very high in general, even in the control condition, so while the benefit of an additional eHMI was there, the degree of success is limited in as far as the shuttle was able to navigate through traffic without the eHMI and the eHMI simply improved by further reducing the number of conflicts. There were also few differences in performance between *RG* and *AN*, which suggested an attention-drawing eHMI on the front of the shuttle being there to be the most important aspect, and the actual design of it being secondary.

### 5.2 Oslo, Norway—eHMI for close and risky overtaking situations (MS2)

Overtaking is a frequently encountered situation with a lot of potential risks associated with it. From a video observation study conducted in Oslo, close and risky overtaking had been identified as one of the most critical scenarios for automated shuttles in mixed traffic Pokorny et al. (2021). Due to the shuttles’ defensive driving style, other road users often decide to overtake the shuttle, even on locations where overtaking is not allowed or under risky circumstances (e.g., oncoming traffic or a limited sight distance). Furthermore, in many of these overtaking maneuvers, the drivers do not keep sufficient distance from the shuttle and finish the overtaking maneuver too close in front of the shuttle. This overtaking behavior can have negative safety consequences, such as abrupt stops of the shuttle when the overtaking road user enters the shuttle’s safety zone or other traffic participants being endangered by the overtaking vehicle.

**FIGURE 16:**
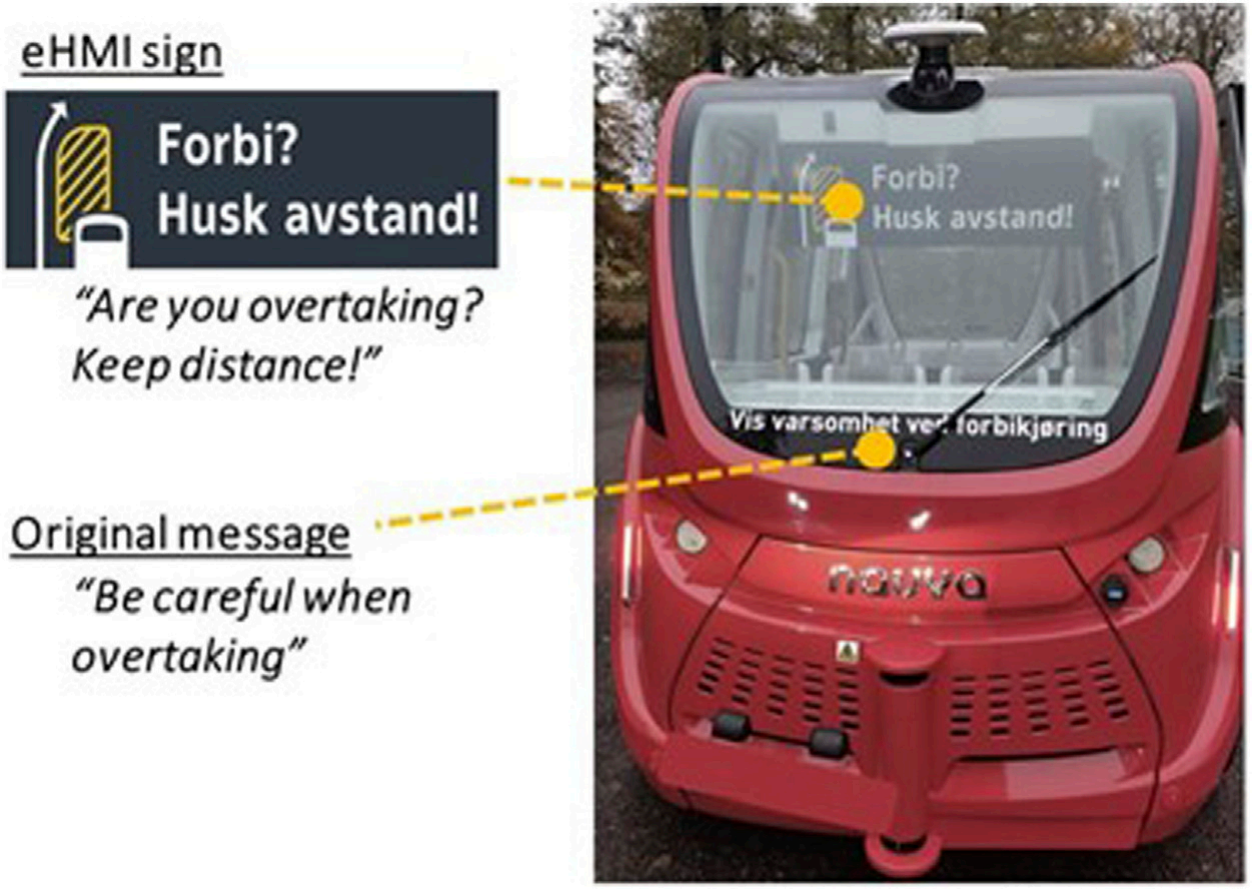
One of the shuttles that roamed Oslo showing the overtaking eHMI.

To mitigate these negative effects, an eHMI was designed specifically to affect the overtaking behavior of other road users in one of the trial projects in the Oslo region, on Ormøya island. Several shuttles (Navya Arma) were operated in 2020 on a 1.3 km long route in suburban neighborhood in mixed traffic, on a narrow curvy public road with a speed limit of 30 km/h. The shuttle had originally displayed the text message “Be careful when overtaking!” on a back display (see Figure 16).

On the basis of the results from the observations, the goal was to strengthen the message with use of an additional eHMI sign and evaluate its effect. The eHMI was conceived at TØI^1^ *via* brainstorming sessions involving several researchers from associated domains (such as traffic psychologists and road safety experts) in order to find out how to convey the message to drivers that they should 1) take care during overtaking and 2) not come too close to the shuttle when overtaking. A decision was made to combine a textual message with a graphic illustration, resulting in a first draft of the eHMI sign. The draft was then iterated with a professional designer from the Oslo public transport provider RUTER who then prepared several design alternatives. The final design (see Figure 16) was selected jointly by the expert panel and the graphic designer. It was then implemented in the shuttle display.

#### 5.2.1 Method

The shuttles drove a total of 1357,4 km over the evaluation period, 269,3 km of which with the eHMI active. In order to assess the eHMIs performance, event data was extracted from the shuttles’ autogenerated logs and roadside interviews were conducted with bystanders. In this publication, the focus will be on the roadside interviews and selected log data related to the shuttle’s detection and braking behavior.

The roadside interviews were conducted as a small scale survey with *n* = 28 respondents.In order to explore whether traffic participants in the area had noticed the eHMI sign and comprehended its meaning, the survey was carried out in December 2020, when the eHMI sign in the shuttle had then been operational for about 1 month. The interviews took place along the shuttles’ route, with people walking along the route or having just parked their car at a parking lot near the beginning of the route. All respondents signed for their consent. The interviewer asked the following questions:

• How often are you in this area?
• Do you have a driving license?
• Are you aware that the automated shuttle drives here?
• Have you ever used the shuttle as a passenger?
• Have you ever overtaken the shuttle (as a driver or a cyclist)?
• If yes, under what circumstances?
• Did the bus somehow react to being overtaken by you?
• Do you know the meaning of this sign?
• What do you think that this sign means?

The interview protocol was setup as an online questionnaire (using QuenchTec software) and the answers were entered with a notebook by the interviewer.

#### 5.2.2 Results

Table 1 shows the sums of events (strong and severe braking, obstacles detected) identified from log data, their frequencies per km driven and the mean frequency of all events per km driven for the eHMI and control conditions. The frequencies per km are provided since the distances traveled by the shuttles was not identical for both conditions (269.3 with the eHMI, 326.3 without).

**TABLE 1:** Events recorded in the shuttle log across both eHMI and control conditions.

Condition	Event	Sum	Frequency (per km)	Mean frequency
eHMI	Strong braking	232	0.86	0.70
	Severe braking	75	0.28	
	Obstacles detected	262	0.97	
Control	Strong braking	391	1.20	0.61
	Severe braking	77	0.24	
	Obstacles detected	127	0.39	

Looking at the individual type of events, there is an evident decrease in frequencies of *strong braking* and an increase of *obstacles detected* in the eHMI condition. For the shuttles without the eHMI sign, the tendency is the opposite in the after period (an increase in *strong braking* and a decrease in *obstacles detected*). As the sum of *strong braking* and *obstacles detected* events is almost similar for shuttles with and without the eHMI sign (494 and 519), we might assume that the number of drivers overtaking the shuttles with and without eHMI sign was about the same. However, their overtaking behavior might have differed, because the reactions to the shuttles differed: If those who were overtaking the shuttles with eHMI finished the overtaking maneuver further in front of the shuttle (an intended consequence of the design), they would more often just be registered as *obstacles detected*, while those who were overtaking the shuttles without eHMI sign would finished the overtaking closer in front of the bus, which led to more frequent instances of *strong braking*.

Results from the interviews show that the vast majority of respondents were familiar with the Ormøya area as they indicated to be there every day (*n* = 24, 86%), they were familiar with the shuttle (*n* = 26, 93%), and had a driver license (*n* = 25, 89%). More than a third (*n* = 10, 36%) had been a passenger on the shuttle. Most respondents reported that they had overtaken the shuttle after driving behind it as a car driver (*n* = 23, 82%), and one had overtaken the shuttle as a cyclist.

When asked in what situation they had driven/cycled behind the shuttle, 21% (*n* = 5) answered that this was while the shuttle was standing still at the stop, 16% (*n* = 4) while the shuttle was driving, and 63% (*n* = 15) in both situations. In most cases the shuttle did not react in any special way when they drove/cycled past it (*n* = 14, 74%). A few respondents answered that the shuttle stopped (*n* = 4, 21%) or braked (*n* = 1, 5%) when they drove or cycled past it.

Most road users recognized the sign and reported to understand its meaning. When asked “Do you understand what this sign means?“, 82% (*n* = 23) understood the meaning of the sign, while 11% (*n* = 2) did not. Two respondents were indecisive. Furthermore, respondents were asked to describe in an open text format how they interpret the sign, or what they think when they see it. A variety of answers were provided, and it appears that they generally do not describe the exact message that was meant to be communicated. Most of the respondents did not directly provide an explanation of what the sign means. This might be due to only five of the respondents experiencing the shuttle changing its behavior (braking or stopping) as a cause of the overtaking maneuver.

Most respondents mentioned more general observations and opinions they associate with the sign. These indicate that the respondents are generally familiar with the fact that it is challenging to overtake the shuttle on this particular narrow road. They mentioned that the shuttle drives slowly, is difficult to overtake, that it is difficult to plan to overtake, and there is often a queue behind the shuttle (“it is difficult to get past the shuttle and it is slow”). A few mentioned that it is “annoying” or “irritating” that the shuttle drives so slow, particularly when there is a heavy vehicle on the route as well, whereas others mention the shuttle is “sweet”, “good for old people”, and that it is “nice and good”.

#### 5.2.3 Summary

Due to the various methodological challenges (e.g. different seasons in before and after periods, lack of good experimental control, the exact reasons for the events identified from log data are unknown, small sample size in the survey) it is difficult to make statistically solid conclusions regarding the effects of the eHMI sign. Therefore, the results should be interpreted with the utmost care. Analysis of log files indicate a positive effect of the eHMI sign on overtaking behavior. From comparison of log data, we see that the frequency of *strong braking* decreased and number of *obstacles detected* (without braking) events increased in the eHMI condition, while opposite trend was found for the shuttles without the sign. This may mean that the drivers overtook the shuttles with the sign more carefully and were just detected as an obstacle, not causing strong braking. The roadside survey shows that the respondents are familiar with the fact that it is challenging to overtake the shuttle on this particular narrow road. Most of respondents believed to understand the message displayed on eHMI sign, however they generally do not describe the exact message that was meant to communicate.

### 5.3 Linköping, Sweden—automated docking co-simulation (MS3)

A study in Linköping, Sweden, investigated the passengers’ experience with automated docking. If buses with automated docking functions are introduced in regular public transport in mixed traffic, there is likely a need for vulnerable road users (VRU) in the surrounding to be informed whether an approaching bus is automated or driven manually. The study, therefore, perfectly supplemented the previously described efforts as well as the same requirements that had been identified in the Digibus project, where there had only been a partially implemented solution (visuals in the doors) and limited validation.

**FIGURE 17:**
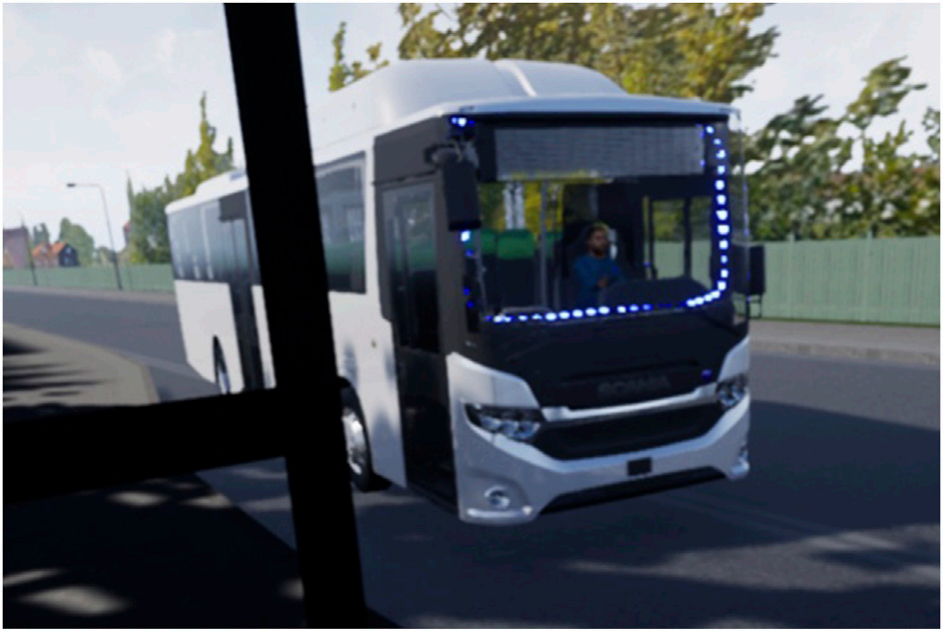
Bus with eHMI active viewed from the outside.

The aim of the study was to develop and evaluate a co-simulation platform where the interaction between automated buses and VRUs could be evaluated. A second aim was to test alternative internal and vehicle-external HMI options for the vehicle–passenger and driver–vehicle interaction at bus transit points in simulated environments. In this study, the results regarding the second aim are reported. The eHMI used consisted of blue lights mounted along the windshield and steering wheel (see Figure 17) for a picture of the actual bus with the eHMI active. A more detailed description of the methods and results can be found in Sjörs Dahlman et al. (2022).

#### 5.3.1 Method

For safety reasons, the interactions between the automated bus and VRUs was evaluated using virtual reality (VR). The specific scenario was a VR/VR co-simulation of an automated docking at a bus stop from both the passengers’ and bus driver’s perspective. The simulation was done using two VR-headsets from HTC; one HTC tobii and one HTC VIVE and each headset had two motion controllers. Both passengers and busdrivers wore headphones to simulate the sound from the bus.

**FIGURE 18:**
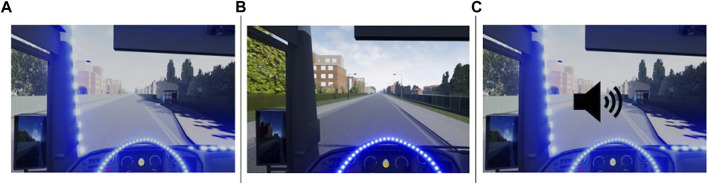
Three eHMI conditions viewed from the inside of the bus: steering wheel and windshield **(A)**; steering wheel only **(B)**; steering wheel, windshield, and sound cue **(C)**.

Three different HMI concepts were tested, system A, B, and C (see Figure 18), with different solutions for communicating information about automation status to the driver and VRUs. System A provided the information by illuminating light strips around the windshield and on the steering wheel. The lights on the steering wheel could not be seen by VRUs outside the bus. Blue lights indicated that the bus was in automated mode and amber lights were used for the handover between manual and automated mode. System B had only the lights on the steering wheel that could not be seen outside the bus and thus looked like a regular bus from the passengers’ perspective. System C had the same features as system A but it also played a bell sound, when the bus approached the bus stop. The sound could be heard outside the bus and the frequency increased as it came closer to the bus stop. The sound was not played inside the bus. Thus, system A and C were the same from the bus drivers’ perspective.

Five bus drivers (one female and four male) and 15 passengers (seven female and eight male) participated in the trial. The age of the passengers ranged from 18 to 60 years and most of them (11 out of 15) were younger than 35 years old. The bus drivers were between 45–60 years old and had at least 2 years of experience in driving buses. The recruitment was done as a convenience sampling of people working or studying at the Linköping University campus area.

For most of the trials, one passenger and one bus driver performed the simulated docking scenarios at the same time as a co-simulation. Since there were fewer bus drivers than passengers, the test leader controlled the bus and acted as a bus driver for some of the passengers. The task for the bus drivers was to drive the bus (manually) between each stop, to hand over the control to the bus when approaching the bus stop, to take back control from the bus after the stop, and to open and close the doors. The passengers’ task was to wait for the bus at each stop and get on the bus and take a seat. Each HMI solution was tested three times, resulting in a total of nine docking scenarios.

The participants’ opinions about the automated docking and the different HMIs were investigated using questionnaires and interviews. The questionnaire included background questions, specific HMI related questions and instruments for measuring trust, acceptance, and usability. The chosen instruments were the Technology Acceptance Questionnaire (TAQ, Van Der Laan et al., 1997), the System Usability Scale (SUS, Brooke, 1996), the User Experience Questionnaire (UEQ, Schrepp et al., 2017), and the SHAPE Automation Trust Index (SATI, Dehn, 2008). The participants answered all questionnaires three times, once for each system. An interview was performed in the end of the test capture the participants’ opinions about the sound in system C and the light in the windshield and on the steering wheel. They were also asked about any suggestions to improve the HMI systems.

The study was planned and conducted according to the Drive2theFuture project’s ethical guidelines. Informed consent was collected from all participants. Since the study was conducted during the COVID-19 pandemic, specific routines to minimize risk of spreading COVID-19 were taken. The questionnaire data were analyzed descriptively and compared between systems using SPSS.

#### 5.3.2 Results

##### 5.3.2.1 Questionnaires

**FIGURE 19:**
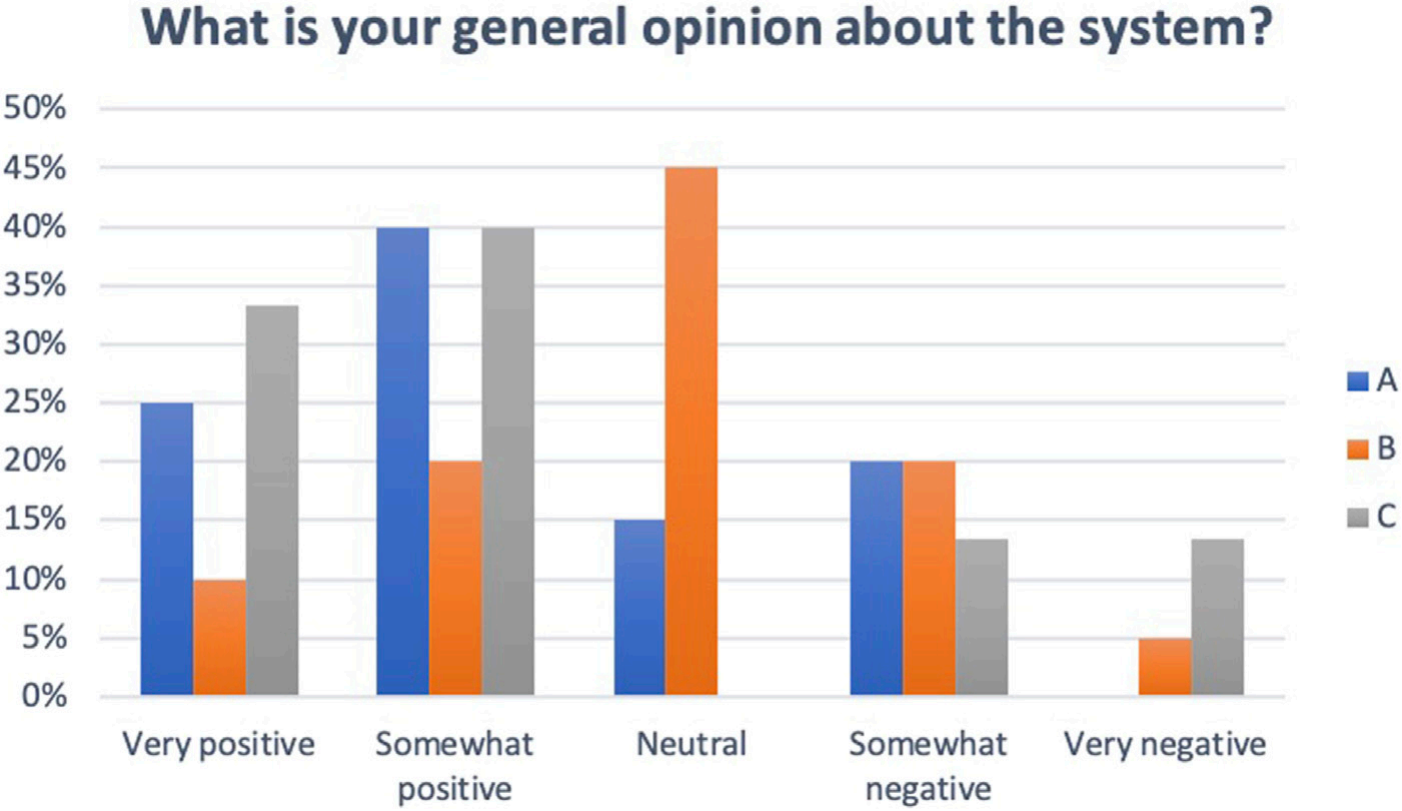
Participants’ general opinion about the eHMI systems.

Figure 19 shows that most participants had a positive opinion about system A whereas the overall view of system B was neutral. System C had more positive than negative ratings, but two individuals rated the system as very negative, indicating that system A had a better overall rating.

**FIGURE 20:**
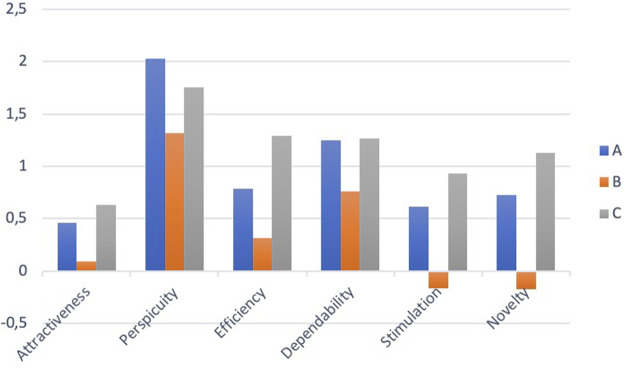
Subscale scores of the UEQ.

System C had the highest usefulness scores on the TAQ. The three systems had similar satisfying scores. Usefulness scores were 0.79 (SD 0.79) for system A, −0.05 (SD 1.12) for system B, and 1.30 (SD 0.74) for system C. Satisfying scores were 0.28 (SD 0.38) for system A, 0.26 (SD 0.35) for system B, and 0.22 (SD 0.74) for system C. System A and C had better SUS ratings on most items but system C was rated as unnecessarily complex by a few participants. The SUS score was 81.4 (SD 15.1) for system A, 77.0 (SD 16.5) for system B, and 82.5 (SD 14.0) for system C. The overall SATI trust scores were: A = 4.9 (SD 0.9), B = 4.1 (SD 1.5), and C = 5.1 (SD 0.8). The user experiences as measured by the UEQ were quite similar for systems A and C whereas system B was rated as less efficient, stimulating and novel (see Figure 20).

**FIGURE 21:**
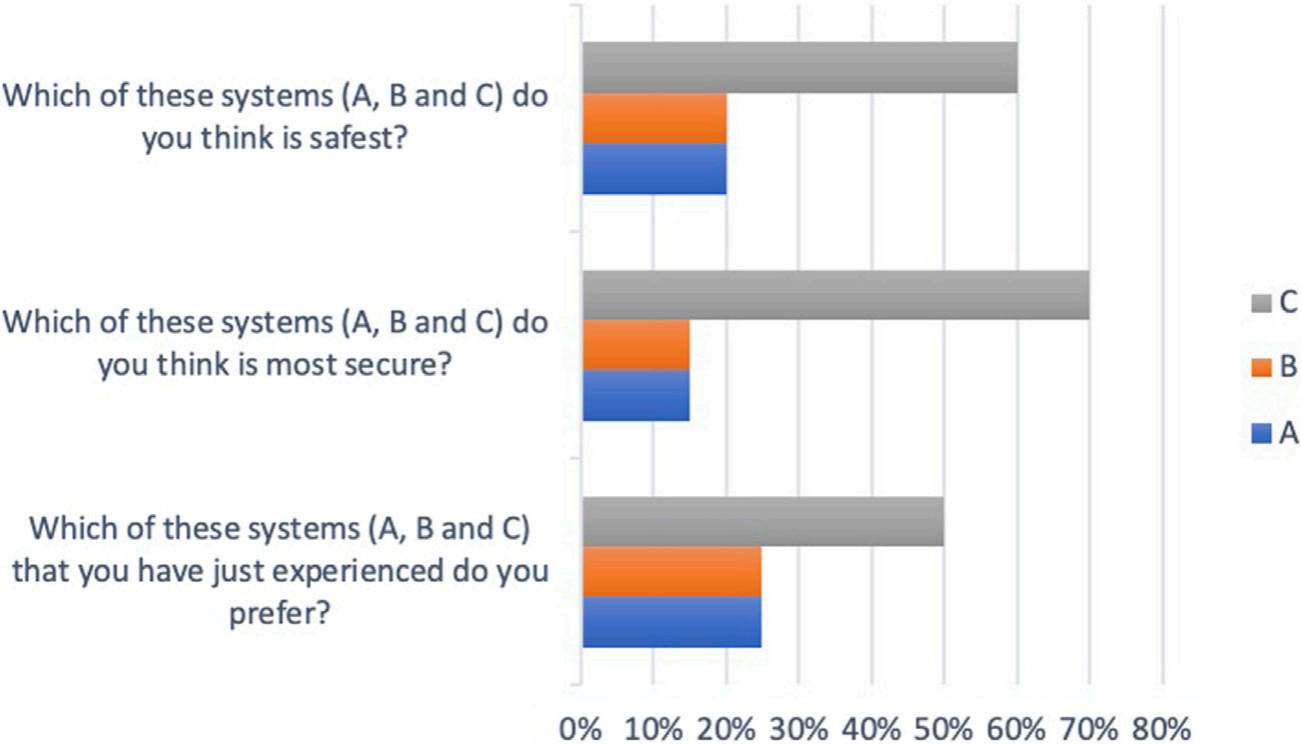
Perceived safety, security, and preference of the three HMI systems.

Specific questions regarding the participants’ opinion about the three HMI systems revealed that system C was perceived as most safe and secure, and it was preferred by most participants (see Figure 21).

**FIGURE 22:**
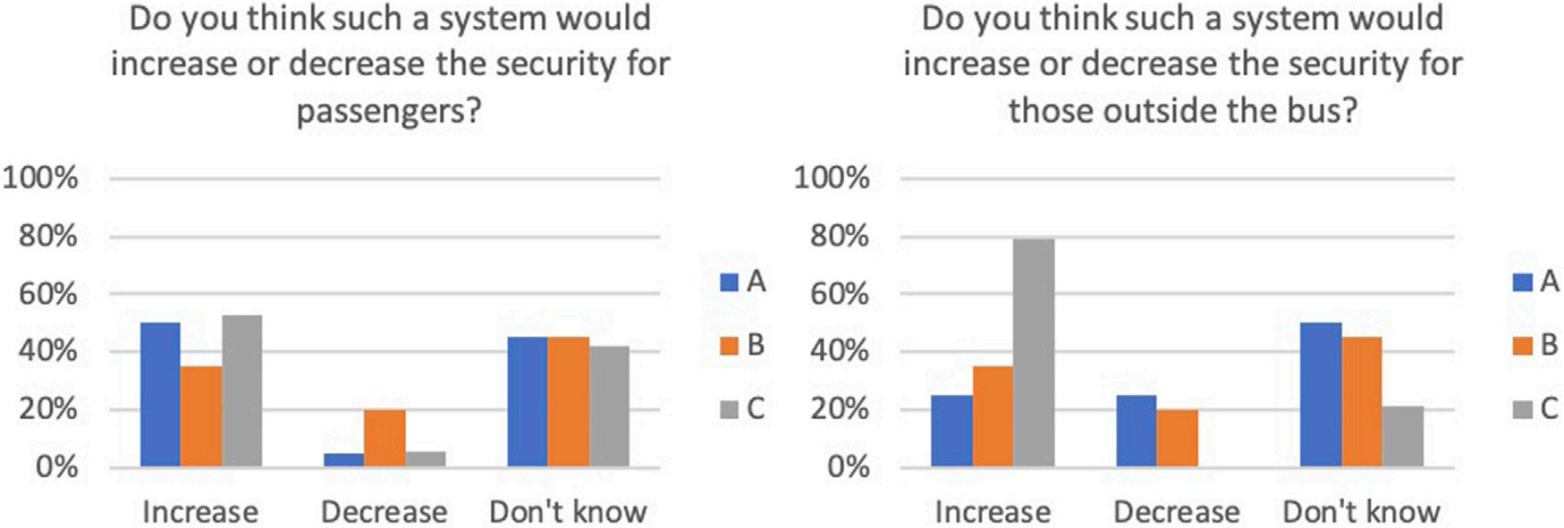
Perceived security outside the bus.

Perceived security for travelers inside the bus and people outside the bus was also rated by the participants (see Figure 22). System C was perceived as the best system regarding security for those outside the bus.

##### 5.3.2.2 Interviews

The passengers expressed that it was important to them to be informed that the bus was automated and most of them thought it should be visible on the bus. The perception of the sound was both positive and negative. The bus drivers expressed that they did not want to be disturbed by any sound. The perception of the light was mostly positive. The participants thought that the purpose of the light and sound was somewhat unclear. They expressed a need for an explanation about the purpose of the light and sound and about the meaning of the different colors. Most passengers did not change their behavior depending on the HMI but some passengers felt more attentive because they did not fully trust the autonomous bus. Suggestions of how to improve the HMI included making the light stronger or making the eHMI more dynamic to communicate if the VRUs were too close to the bus. There were also suggestions to include information about automation status at the bus stop.

#### 5.3.3 Conclusion

The results of the HMI evaluation showed that system C (sound and lights in the windshield and on the steering wheel) and system A (lights in the windshield and on the steering wheel) were rated as more useful than system B (light on steering wheel only) on the TAQ. System C was also rated with highest scores on the SUS and SATI. However, when it came to the general opinion about the system there were no major differences between A and C. From the interviews it was evident that passengers prefer to know if the bus is in automated mode or not when it approaches. The participants expressed a need for instructions or training on what the different components of the HMI are intended to communicate.

### 5.4 Vienna, Austria—eHMI location and modality during passenger changeover (MS4)

Managing passenger turnover through eHMIs was also investigated in an experiment of the auto.Bus—Seestadt. The focus was on two eHMI application scenarios of passenger turnover situations: information on that the bus is about to start (start announcement), and an indication when too many passengers are in the bus (capacity limit). The questions were as follows:

• Which modalities of presentation are conceived as supportive by passengers (audio, animation, screen with icon and text)?
• Where should eHMI be located (on the shuttle, at the station, or on a wearable, such as a smartwatch)
• Do preferences about eHMI presentation modality and location differ between usage scenarios, namely start announcement vs. capacity limit?

#### 5.4.1 Method

**FIGURE 23:**
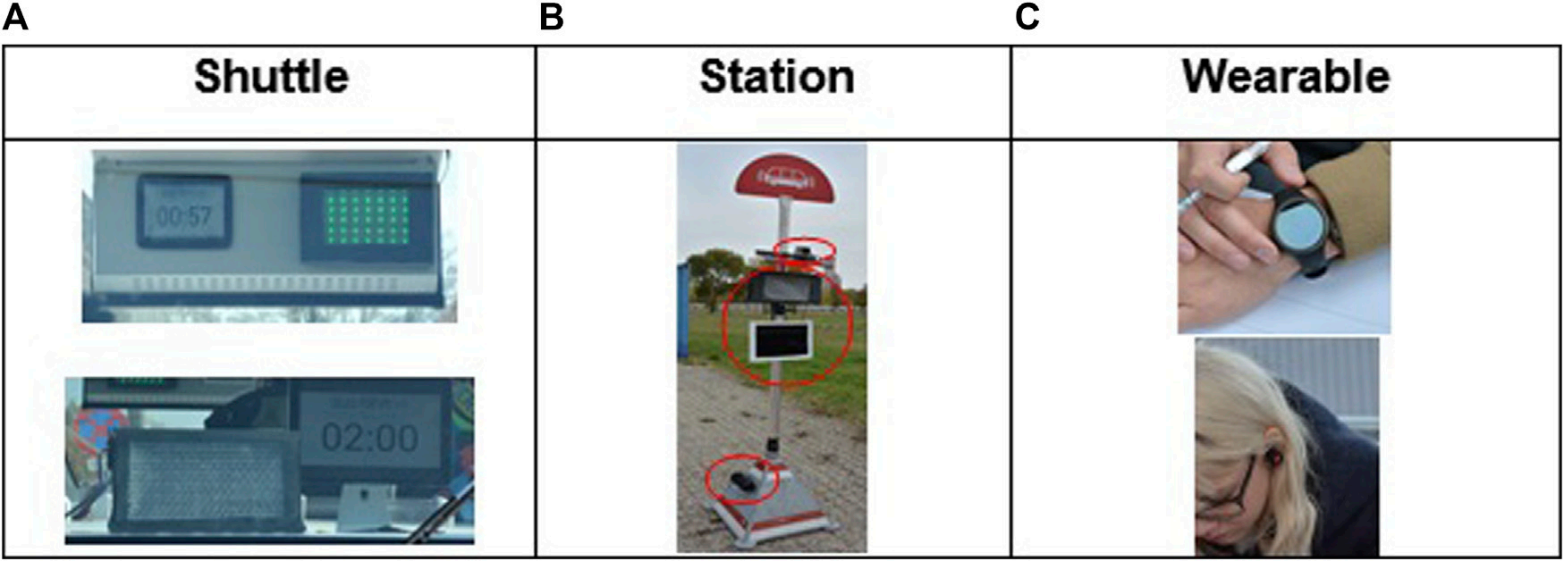
**(A)** Locations of screen and animations (LED array) on the bus interior facing inwards (picture at the top) and behind the windscreen facing outwards (bottom); the loudspeaker was mounted below the seats; **(B)**: locations of color animation (LED display at the top), screen (tablet in the middle), and loudspeaker (bottom); **(C)**: wearable (smartwatch) at the wrist (top) and bluetooth earplugs (bottom).

To investigate the questions mentioned above, an experimental study with an automated shuttle in a protected area for intent communication displays was conducted. 31 participants, with a mean age of 45 years (from 25 to 66 years) and a balanced gender distribution (16 male, 15 female) were invited to the study. There were two experimental factors: eHMI location (on the bus, at the station, on the wearable), presentation modality (information screen, animation, audio) and an intermittent variable representing the two above mentioned passenger changeover scenarios. The combination of these three factors resulted in 9 different experimental conditions. Figure 23 shows the prototype realizations of these nine combinations. The test prototypes were operated using a Wizard of Oz setup.

Participants were welcomed and briefed about the study. They were then confronted with the nine test situations, each of which consisted of a combination of one presentation type and one presentation modality. In the test situations, participants waited for the shuttle arriving at the station, then entered the shuttle, sat down, rode to the next test station, and stepped out of the shuttle. During this process, they were exposed to the eHMI communication of the two scenarios (start announcement and capacity limit). After having experienced all of these situations, they were asked to fill in a survey in which they indicated which of the combinations they would like to be implemented for realizing the two scenarios.

#### 5.4.2 Results

**FIGURE 24:**
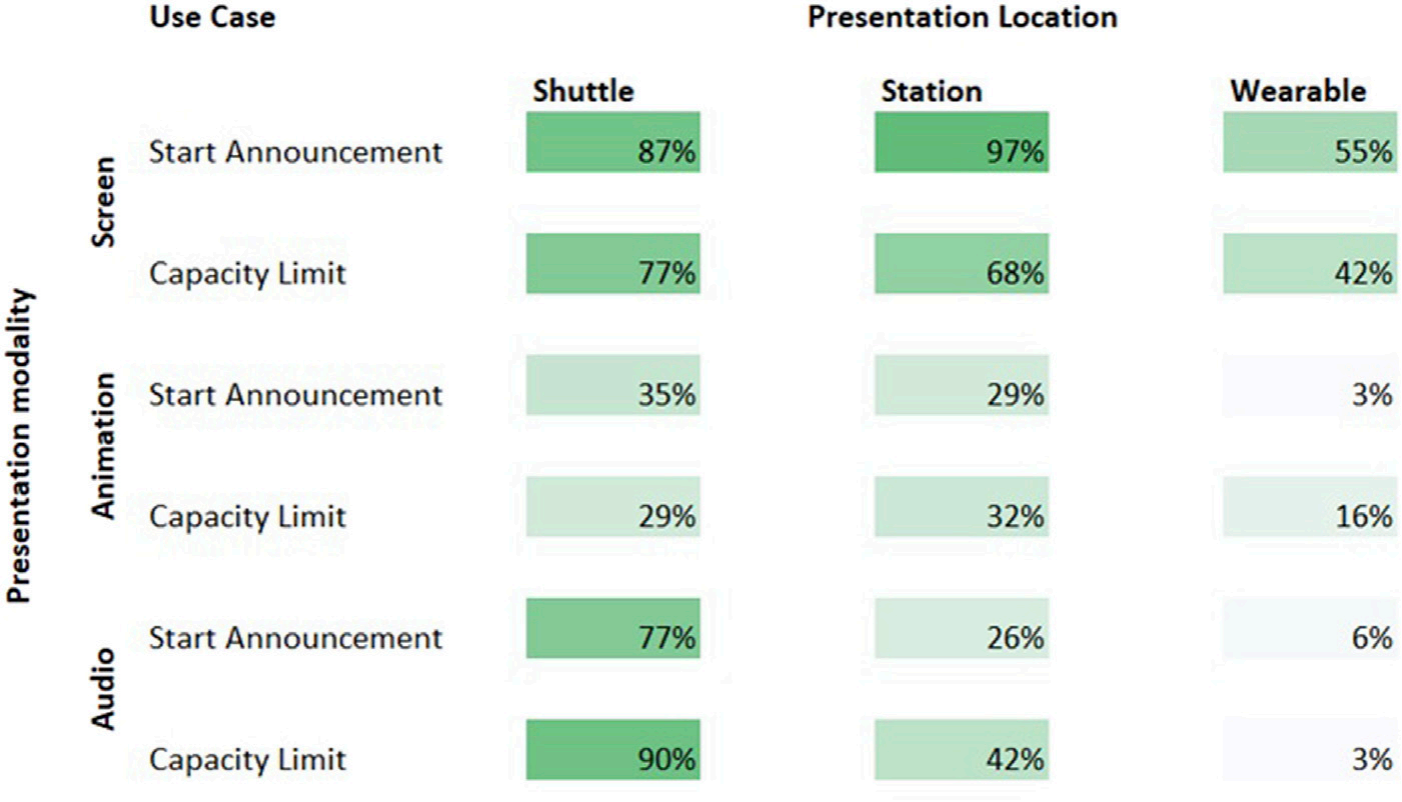
Preference for each combination of modality and location in each of the two investigated scenarios. The percentages are the ratios of participants’s answers whether or not they would like to see the eHMI variant in the respective scenario.

In general, for both investigated scenarios—the countdown information when waiting for the start of the bus and the indication on an overfilled bus—would benefit from eHMIs. However, as Figure 24 indicates, there were different patterns of preference, across the investigated factors. Providing the information on wearables was least preferred, especially when communicated through animations or audio. eHMIs mounted on the shuttle itself was most preferred, but not when provided through dynamic animations. Presenting information at the bus station was only wished when provided by a screen, especially when information on entering the bus was to be communicated. In general, animations were least preferred, screens with text and iconic information were the most preferred display for the station and the wearable. For eHMIs mounted on the bus, audio and screen were similarly high. Presenting audio for indicating an overfilled bus received the highest preference among all combinations.

## 6 Discussion

In the following, we discuss a number of salient points that arose across the reported studies.

### 6.1 The right eHMI for the right situation

The situations where an eHMI can assist an automated shuttles’ interaction are manifold, involving different stakeholders and other contextual variables. One main distinguishing characteristic is the recipient of information. Here, a difference in available time budget and resulting suitability of different eHMIs could be seen especially in regard to pedestrians vs. motorized road users: Pedestrians generally have a larger time budget available. As a result, more complex visualizations, including verbal information and extended animations, can be both useful and appropriate.

Conversely, for motorized road users, the shuttle is typically only one of many other external factors that a driver has to pay attention to. The time budget is very limited as a consequence and any driver’s gaze cannot be expected to remain on the shuttle for long. Therefore, shorter, non-verbal cues are more appropriate here with visibility/noticeability having priority over richness of communicated content.

Despite contrary initial results in the preparatory studies, one-dimensional LEDs proved moderately effective for both shuttle-to-vehicle interaction (MS1) as well as shuttle-to-pedestrian (MS2) interaction. The most likely explanation for this are the contextual influences of weather and resulting different light conditions as well as the many different angles of approach of the other road users. A well-designed display that can’t be seen or fully comprehended under most circumstances is ultimately less useful than a simple interface that is visible. When the circumstances can be controlled, such as when the message is always targeted in a specific direction (behind or in front on the same lane, or at the doors at the bus stop), then a high-resolution display containing even verbal information can be effective, as MS3 showed. Thus, the less controlled the interaction in terms of communication is, the simpler the eHMI cue has to be.

Finally, the shuttle’s communication need not only encompass eHMIs on the shuttle (MS4). Pedestrians waiting at a bus stop do not necessarily need to wait for the shuttle to arrive in order to receive boarding-relevant information, reducing the need for additional on-shuttle eHMIs and resulting potential clutter or interference with other indicators. While the potential to use such solutions also for shuttle-to-vehicle-communication is limited, integration with traffic lights (both for pedestrian as well as vehicle crossings) is a possibility, provided the shuttle is connected to the infrastructure. Beyond that, wearables, while unlikely to be able to serve as a replacement, can provide additional assurance. For vehicle interaction, additional information could also be part of the vehicle’s UI, although that would, once again, require the shuttle to be connected.

### 6.2 Subjective preferences vs. objective effects

A view on the results across all studies revealed a difference between the perceived subjective preferences and the objective effects of having an AV equipped with an eHMI. As suggested in the literature (Löcken et al. (2019); Kaleefathullah et al. (2020); Kooijman et al. (2019); Velasco et al. (2019); De Clercq et al. (2019); Rettenmaier et al. (2020); Faas et al. (2021)), eHMIs could be confirmed to often have positive effects on users’ interactions with AV when it came to subjective ratings. There, differences to interaction without an eHMI were clearly visible with a considerable difference between control and eHMI conditions.

The objective assessments, on the other hand, showed more modest differences in performance. The video observations conducted in Oslo (MS3) did not show decisive results regarding a possible safety increase when the eHMI was active. Similarly, the field tests in Austria (MS1) showed rather modest performance increases of the eHMI over the control condition. The reasons for this discrepancy between subjective and objective results are difficult to identify, since there were several factors that influenced the field assessments: Different weather conditions meant different visibility conditions across assessments. In addition, while the interactions to be observed were pre-defined (overtaking in Oslo, intersections and crossings in Austria), the vehicle speeds, angles of approach, and distances between shuttle and other vehicles were varied, leading to further heterogeneity within the interaction scenarios, even when conceptually similar.

A further aspect to be considered is that, by necessity, the baseline safety across all conditions has to be high for a shuttle to be able to be deployed in public traffic (including but not limited to the shuttles driving at very low speeds). Since the eHMIs are primarily supposed to address safety concerns, any possible performance increases can only occur on the upper spectrum of making a sufficiently safe interaction potentially even safer. Still, without a clear indication of the exact source of the discrepancies, these results would support the position that implicit communication *via* the vehicle’s behavior is a stronger influencing factor on safe interaction than the presence of any additional eHMIs (see Löcken et al., 2019; Kaleefathullah et al., 2020; Kooijman et al., 2019; Velasco et al., 2019; De Clercq et al., 2019; Rettenmaier et al., 2020; Faas et al., 2021; Moore et al., 2019).

### 6.3 Lab vs. field and the limits of both

Due to the lack of ecological validity, studies in virtual or online contexts are only partially suitable for evaluating the usability of eHMI in its entirety. While individual aspects such as colors or positioning can be tested, the experience of actual traffic situations is only possible to a limited extent, and insights gained may not prove reproducible in a real-world context. This is not a novelty in any way and part of standard scientific knowledge, which is why validation is usually eventually sought in field tests after initial laboratory or otherwise simulated settings.

What makes this a point particular to studying interactions with AV are the necessary constraints of any field trial involving them. As mentioned in the previous section, the interaction cannot be dangerous for the participants in any realistic degree or the study must not be conducted. By necessity, some aspects then have to be simulated even within the field setting or contextual variables have to be modified to reduce the risk level appropriately (e.g., driving at very low speeds). This generally limits the ecological validity of the results obtained, as the solutions are intended for a different (higher risk) context than they are evaluated in.

Also, adapting to a study context requires a cognitive effort on the part of the participants, which can be more or less challenging depending on the study setup. While VR technology offers the possibility to move safely in space and to interact with vehicles and traffic situations more realistically than is possible in an online questionnaire, the cognitive effort to familiarize oneself with the technology, operation, and aesthetics of the world is significantly higher. Symptoms of nausea and dizziness may occur. Field studies or online surveys, on the other hand, require less adaptation effort from the participants. Online assessments, however, do not achieve nearly as good a degree of realism or as high a level of involvement, while, as mentioned, in field studies certain situations cannot be reproduced due to safety aspects.

Thus, while it is difficult to prescribe an exact recipe here, we do conclude from the learnings across all studies that for a safety-relevant technology such as AV, simulations and controlled laboratory results can and should play a role to supplement field trials even within the final phases of validation.

## 7 Conclusion

In this study, we presented eHMI solutions and evaluation study results from three collaborating research projects in Europe: The Austrian flagship projects *Digibus® Austria* and *auto.bus Seestadt*, as well as the European Horizon 2020 project *Drive2theFuture*.

The preparatory activities opened a wide spectrum of critical interaction scenarios, ranging from pedestrian crossings, which are well-covered by existing approaches, to intersections with other road users, boarding and docking operations, as well as dangerous passing and overtaking situations, all of which are less covered by existing approaches.

Across all activities we found a difference between subjective and objective performance of eHMIs, where the subjective gain would be higher than the objective one regarding safety. This is due the situations being already rather safe than unsafe but also the constraints of the field context, where risks must be minimized as part of the study preparation, so any safety gain can only occur in the upper spectrum.

The discrepancy was also found in terms of simulated vs. field performance, where animations and verbal information, especially for shuttle-to-vehicle-communication, did not perform as well in the field due to contextual influences. Overall, simple light-based indicators were found useful both for crossing situations with motorized road users and docking operations (pedestrian/passenger communication) due to good visibility under multiple angles and weather conditions and the required information density being rather low in these circumstances. Dangerous overtaking—and by extension other interaction situations where the angle of approach and viewing the eHMI can be controlled—can be addressed *via* more high-resolution information, including verbal content. Finally, interaction at the bus stop, especially regarding itinerary and capacity management, need not be limited to eHMIs on the shuttle only, with smart station displays or even wearables sensibly extending both the physical and temporal reach of the shuttle.

## Data Availability

The datasets presented in this article are not readily available because sharing of datasets requires agreement of the respective consortia, which needs to be negotiated with the respective contributing authors. These decisions can be mediated but not fully made by the corresponding author alone. Requests to access the datasets should be directed to alexander.mirnig@plus.ac.at.

## Footnotes

1
https://www.toi.no/
